# Calcium-based synaptic and structural plasticity link pathological activity to synaptic reorganization in Parkinson’s disease

**DOI:** 10.1126/sciadv.adw7421

**Published:** 2025-11-07

**Authors:** Cathal McLoughlin, Justus A. Kromer, Madeleine Lowery, Peter A. Tass

**Affiliations:** ^1^ Department of Electrical and Electronic Engineering, University College Dublin, Dublin D04 V1W8, Ireland.; ^2^ Department of Neurosurgery, Stanford University, Stanford, CA, USA.

## Abstract

Motor symptoms of Parkinson’s disease (PD) are associated with dopaminergic neuronal loss. Widespread synaptic reorganization and neural activity changes, including exaggerated beta oscillations and bursting, follow dopamine depletion (DD) of the basal ganglia (BG). Our computational model examines DD-induced neural activity changes and synaptic reorganization in the BG subcircuit comprising the subthalamic nucleus and globus pallidus externus. Calcium-dependent synaptic and structural plasticity mechanisms were incorporated, allowing neural activity to alter network topology. We show how hyperactivity of indirect pathway striatal projection neurons (iMSN) can induce synaptic connectivity changes consistent with PD animal models. Our results suggest that synaptic reorganization following DD results from a series of homeostatic calcium–based synaptic changes triggered by iMSN hyperactivity. While this structural plasticity functions as a compensatory mechanism in the cascade of changes following elevated iMSN input from striatal DD, it may become compromised if iMSN and cortical inputs show substantial bursting activity.

## Teaser

Synaptic and structural plasticity differentially drive synaptic reorganization and abnormal activity in Parkinson’s disease.

## INTRODUCTION

### Symptoms, causes, and pathways of PD

Parkinson’s disease (PD) is a slowly progressing neurodegenerative disorder characterized by both motor symptoms (such as tremors, rigidity, akinesia, and bradykinesia) and nonmotor symptoms [such as apathy and sleep problems (*1*)]. As the second most common neurodegenerative disorder, PD affects approximately 2 to 3% of people 65 years and older (*2*).

**Fig. 1.**
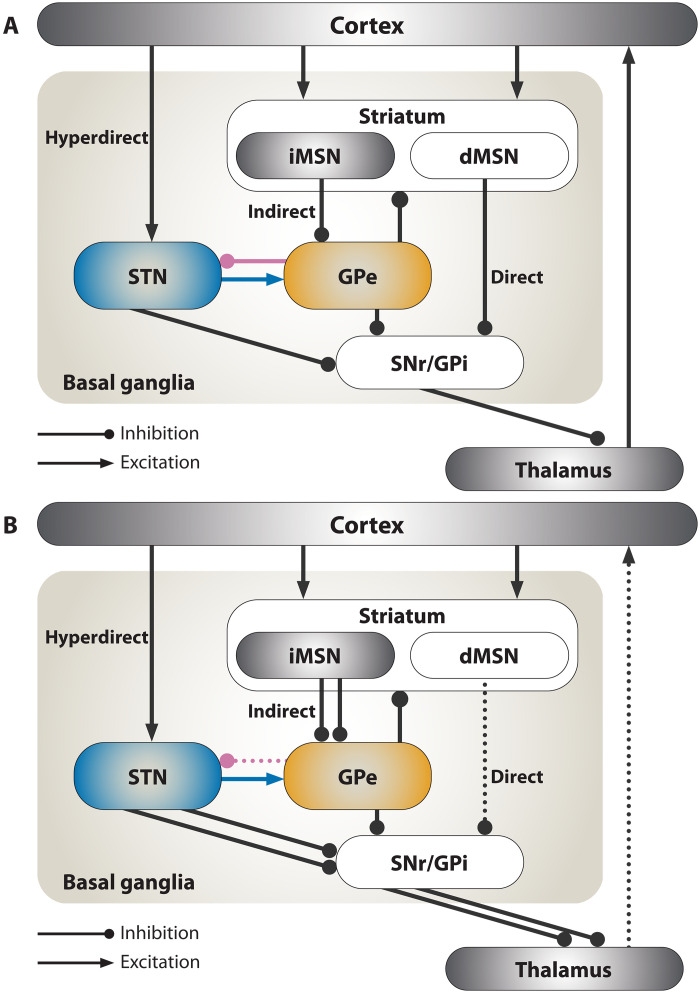
Schematic diagram of synaptic connectivity in the BG. (**A**) According to the direct/indirect pathway model of PD, DD leads to an imbalance between the direct cortex-dMSN-substantia nigra pars reticulata (SNr)/GPi-thalamus pathway and the indirect cortex-iMSN-GPe-STN-SNr/GPi-thalamus pathway (*4*, *5*). Later, the akinetic hyperdirect pathway was identified (*11*). Intra-nucleus connections are not shown. (**B**) Changes in the BG network in PD according to the direct/indirect pathway model (*4*, *5*). Dotted arrows represent weakening, and doubled arrows represent strengthening, relative to the healthy state. Note that STN → GPe, GPe → GPi, GPe → STR, and direct cortical (CTX) → STN connections were not included in the original model (*4*, *5*).

PD motor symptoms appear following the loss of midbrain dopaminergic neurons, resulting in dopamine depletion (DD) in the basal ganglia (BG); however, the exact mechanisms that lead to PD motor symptoms are still undetermined (*3*). In the late 1980s, Albin *et al.* (*4*) and DeLong (*5*) hypothesized a direct/indirect pathway model of BG functionality. The model suggested that BG disorders, including PD, result from an imbalance between the prokinetic direct and the akinetic indirect pathways. The loss of dopaminergic striatal inputs differentially affects indirect pathway (iMSNs) and direct pathway striatal medium spiny projection neurons (dMSNs), resulting in the dominance of the indirect pathway over the direct pathway (*6*). This results in elevated iMSN activity, increased inhibition of the external globus pallidus (GPe), and disinhibition of the subthalamic nucleus (STN) (*7*). Ultimately, this increases the firing rates of BG output neurons, e.g., in the internal globus pallidus (GPi), and over-inhibits thalamocortical motor circuits (Fig. 1, A and B) (*4*, *5*).

Empirical tests of the direct/indirect pathway model in animal models of PD confirmed that bilateral excitation of iMSNs elicited hypokinetic motor symptoms such as freezing and bradykinesia, stressing the critical role of elevated iMSN activity in PD. In contrast, excitation of dMSNs reduced freezing and increased locomotion (*8*). Despite these observations, the delineation of striatal outputs into orthogonal direct and indirect pathways was shown to be less distinct than initially supposed (*6*). Furthermore, the relationship between BG output activity and motor symptoms is inconsistent (*9*, *10*). Specifically, the expected increase of substantia nigra pars reticulata activity in PD was not observed (*9*). Furthermore, suppression of GPi activity did not promote motor activity in monkeys (*10*).

### Pathological neural activity and synaptic reorganization

Later, it was suggested that pathological activity patterns, such as bursting and exaggerated synchronous oscillations, underlie PD motor symptoms by preventing individual neurons from independently processing motor-related information (*3*). The STN-GPe circuit lies at the convergence of the hyperdirect (*11*) and indirect pathways (Fig. 1); it is a crucial area where exaggerated beta oscillations manifest (*12*–*15*) and therapeutic deep brain stimulation (DBS) is applied in PD (*16*). Beta oscillations were also observed in healthy control animals (*17*, *18*), and exaggerated beta oscillations in animal models of PD emerge after early motor symptoms, challenging a causal relation to motor symptoms. The latter suggests that exaggerated beta oscillations develop because of slow adaptation processes in related brain networks (*19*–*22*).

**Fig. 2.**
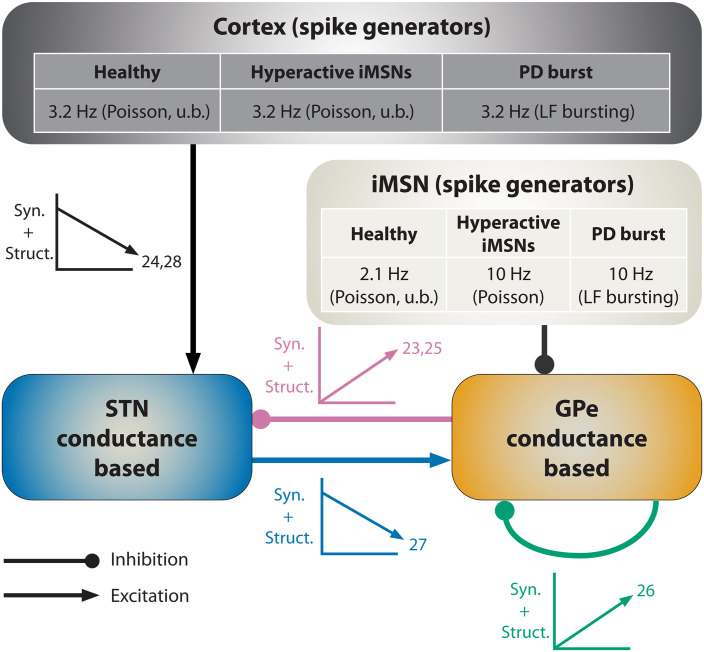
Schematic diagram of our computational model of the STN-GPe circuit. Cortical and iMSN inputs were approximated by Poisson spike generators, with a time-dependent spike rate adjusted to mimic uncorrelated bursting (u.b.) in the healthy state, and two PD states: one with elevated but fixed iMSN Poisson rate parameters and similar CTX activity as in the healthy state (hyperactive iMSNs) and one with time-dependent cortical and iMSN Poisson rates mimicking correlated low-frequency bursting (PD burst.) (See Materials and Methods for details). STN and GPe neurons were described by conductance-based point neuron models previously published in (*46*) and (*45*). Labels next to synaptic connections indicate which types of plasticity were considered with “syn.” referring to calcium-based synaptic plasticity implemented according to Graupner and Brunel (*48*) and “struct.” structural plasticity, which we modeled using a linearized version of the Butz and Van Ooyen framework (*49*). Small arrows indicate whether an increase or a decrease in overall synaptic inputs was observed in PD animal models, respectively (see corresponding references).

Synaptic reorganization, including changes in synaptic strengths and/or numbers, has been proposed as a mechanism contributing to such processes (*22*). Recent experiments in rodent models of PD reported substantial synaptic reorganization in the BG following DD. DD was shown to change the strength of synapses (synaptic plasticity) and the number of synaptic connections (structural plasticity) between several populations of BG neurons (*23*–*29*). Known synaptic changes in the STN-GPe circuit encompass both excitatory and inhibitory synaptic connections (Fig. 2) and likely affect network dynamics, that is, their ability to generate oscillatory activity (*14*, *30*–*32*).

Multiple experimental and modeling studies have produced conflicting results regarding the role of beta oscillations in PD motor symptoms (*19*–*21*, *33*–*35*); in addition, the interplay of pathological neural activity, including hyperactivity of the indirect pathway, bursting, and exaggerated oscillations, and synaptic reorganization is unclear (*22*). Therefore, we chose to examine the interplay of indirect pathway hyperactivity, bursting, and synaptic reorganization in the STN-GPe circuit as a primary aim and its relation to beta oscillations within this circuitry as a secondary aim in our study.

### Brain stimulation–induced synaptic reorganization may contribute to stimulation-outlasting therapeutic effects

The ability to induce positive therapeutic effects that outlast the stimulation period may reduce the overall required stimulation power and the risk of unwanted side effects from current treatments, such as high-frequency DBS (*16*). Coordinated reset stimulation (CRS) (*36*) is a promising computationally developed technique that extends therapeutic effects beyond the initial stimulation. Studies conducted using neuronal network models incorporating synaptic plasticity predicted stimulation-outlasting effects (*36*). In plastic networks, stable synchronized states (mimicking synchronized pathological oscillations in PD) and stable desynchronized states (mimicking the absence of synchronized pathological oscillations) can coexist. During CRS, synaptic connectivity changed, which drove the networks from a synchronized state into a stable desynchronized state, inducing stimulation-outlasting desynchronization effects (*36*). Preclinical studies in the nonhuman primate PD model showed that CRS of the STN induced corresponding cumulative and long-lasting desynchronization and therapeutic effects that outlasted stimulation by several weeks (*37*, *38*). CRS was successfully delivered to patients with PD using DBS electrodes (*39*) and vibrotactile fingertip stimulation (*40*). However, only recently has CRS-induced structural plasticity been studied computationally in the STN-GPe circuit (*41*); however, in this study, only excitatory connections were plastic, and synaptic and structural plasticity were not simulated together (*41*). Recently, studies in DD mice also reported stimulation-outlasting therapeutic effects after inhibition of one type of GPe neurons (*42*). On the basis of these results and the observation that different types of GPe neurons responded in opposite ways to the onset of high-frequency electrical stimulation, Spix *et al.* (*43*) developed a DBS-burst stimulation technique that led to cell-specific responses in brain slices and to stimulation-outlasting therapeutic effects in DD mice. The ability to model synaptic reorganization in the STN-GPe circuit during stimulation targeting stimulation-outlasting and cumulative effects, such as CRS and pallidal burst stimulation (*44*) may help develop clinically testable hypotheses about effective stimulation parameters as well as underlying mechanisms.

### A computational model to examine synaptic reorganization in the STN-GPe circuit

We suggest that synaptic reorganization following DD results from a series of homeostatic calcium–based synaptic changes triggered by elevated iMSN activity. The critical role of elevated iMSN activity in PD pathology presents a core tenet of the direct/indirect pathway model and has been shown experimentally (*8*). However, a model of long-term synaptic plasticity in the BG that explains the observed synaptic changes after chronic DD has not been presented to date. A computational model, in which the relationship between observed behavior and neuronal properties can be directly compared, may help develop future therapeutic strategies that induce synaptic reorganization to promote long-term effects.

We developed a computational model of calcium-based synaptic and structural plasticity in the STN-GPe circuit. The STN is one of the main target regions for high-frequency DBS (*16*) and CRS in PD (*39*) and is believed to be involved in the generation and/or maintenance of exaggerated beta oscillations in PD (Fig. 2) (*13*, *14*, *45*). In particular, we model the calcium concentrations in local dendritic segments and their modulations due to pre- and postsynaptic activity. To achieve the performance necessary for multiday simulations with submillisecond integration time steps, we implemented the model using the GPU parallelization library CUDA.

Our model not only reproduces currently available data on long-term plasticity in the STN-GPe circuit but also demonstrates that calcium-based synaptic and structural plasticity may contribute to the observed synaptic reorganization presenting homeostatic adaptation to counteract hyperactivity of the indirect pathway—a core tenet of the classical direct/indirect pathway model (*4*, *5*). Our findings suggest that indirect pathway hyperactivity (*4*, *5*), as implemented by fast-spiking iMSNs, is sufficient to explain the observed structural synaptic reorganization in PD animal models in the presence of calcium-based synaptic and structural plasticity. However, in our model, indirect pathway hyperactivity alone is not sufficient to reproduce the experimentally observed increases in STN and GPe beta power. Only when we considered correlated CTX and iMSN bursting, in addition to high iMSN firing rates, did our model not only demonstrate synaptic reorganization but also exaggerate STN and GPe beta oscillations.

Structural plasticity led to similar changes in synaptic connectivity regardless of the presence of correlated bursting. Conversely, synaptic plasticity induced opposite shifts in synaptic efficacy in these cases. Furthermore, synaptic and structural plasticity each influenced exaggerated beta oscillations in the STN-GPe circuit differently. When compared to simulations without plasticity, synaptic plasticity alone increased beta power, while structural plasticity alone reduced beta power at low correlated bursting, but at high bursting levels, it had little to no effect or very slightly increased beta power.

We combine existing computational models of STN and GPe neurons with detailed computational models of calcium-based synaptic and structural plasticity to better investigate the relationship between BG neural activity patterns and synaptic reorganization. By doing so, we hope to facilitate the refinement of current therapies and the development of previously unidentified approaches to treating PD.

## RESULTS

### Development of the computational model

We developed a computational model of long-term plasticity in the STN-GPe circuit consisting of 250 STN and 750 prototypic GPe neurons simulated using modified conductance-based models (*45*, *46*). Synapses were represented using the multistate model by Rubin *et al.* (*47*). Excitatory synapses contain AMPA and *N*-methyl-d-aspartate (NMDA) receptors, while inhibitory synapses contain γ-aminobutyric acid type A (GABA_A_) receptors. Acute changes in calcium concentrations alter synaptic conductances, which we model using the framework from Graupner and Brunel (*48*). Their model uses differing calcium concentration thresholds; below a minimum threshold, there is no change in synaptic efficacy, whereas above this threshold, synaptic depression occurs. At high calcium concentrations above an upper threshold, synaptic potentiation occurs.

In addition, we implement homeostatic structural plasticity to maintain an average neural calcium concentration, following the work of Butz and Van Ooyen (*49*). Striatal and cortical inputs are simulated with Poisson spike generators, and their activity is manipulated to study synaptic reorganization in response to changes in striatal and cortical inputs mimicking those observed in PD. These activity-dependent changes of synaptic connectivity result from a combination of voltage-gated calcium channels, dendritic calcium influx through NMDA receptors, and calcium-based synaptic and structural plasticity.

The nature of plasticity at synapses onto STN neurons has been investigated experimentally (*23*, *50*). Plasticity at CTX-to-STN synapses is dependent on pre- and postsynaptic activation. Optogenetic stimulation of cortical axons can induce long-term potentiation (LTP) of CTX-to-STN synapses; LTP is mediated by calcium influx into postsynaptic STN neurons via NMDA receptors (*23*). GPe-to-STN synapses are potentiated concomitantly, demonstrating that plasticity in the STN is heterosynaptic (*51*). Copotentiation of CTX-to-STN and GPe-to-STN synapses appears to balance the levels of excitation and inhibition within the STN neurons (*23*) and thus may act to maintain STN activity at some physiological set point.

In its default state, the model was tuned to reflect the dopamine-intact STN-GPe circuit of the rat, for which the neural firing patterns and rates are well-described in the literature (*52*, *53*). The results demonstrate that our computational model reproduces known characteristics of synaptic plasticity in the STN-GPe circuit, including heterosynaptic plasticity in the STN (*23*, *50*) and long-term plasticity induced by rebound bursting (*50*).

### Heterosynaptic plasticity in the STN

**Fig. 3.**
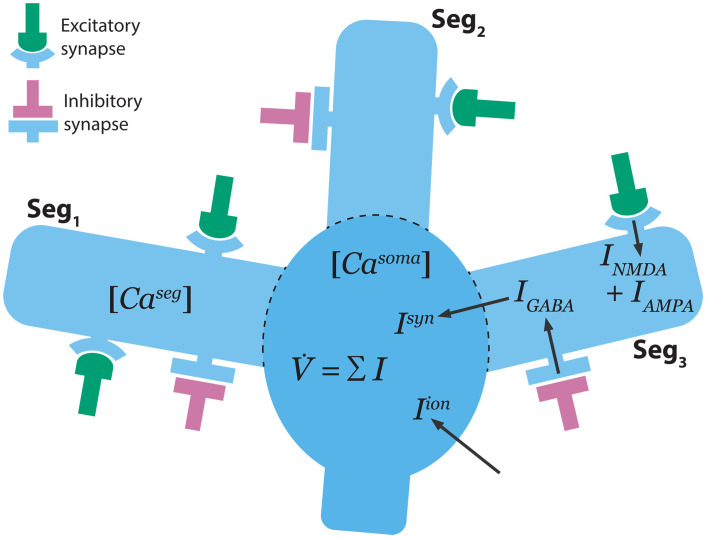
Schematic diagram of a single model neuron in this study. Each neuron contains three isolated segments (Seg_1_, Seg_2_, and Seg_3_), where local calcium concentrations (Ca^seg^) are modeled. These local calcium concentrations influence the efficacy of the excitatory and inhibitory synapses that are present on the segment. Currents contributing to changes in membrane potential, $V_{i}$ , arise from voltage-gated ion channels, $I^{\text{ion}}$ (Table 1), excitatory ( $I^{\text{NMDA}}$ and $I^{\text{AMPA}}$ ), and inhibitory ( $I_{\text{GABA}}$ ) synapses ( $I^{\text{syn}}$ ). Currents contributing to changes in segmental and somatic calcium concentrations arise from T-type ( $I_{T}$ ) and L-type ( $I_{L}$ ) calcium channels, and from the calcium component of NMDA receptors on excitatory synapses ( $I_{\text{NMDA},\text{Ca}}$ ). Segment calcium concentrations are influenced by their local NMDA currents, whereas somatic calcium concentrations are influenced by the NMDA currents from all synapses on the neuron.

**Fig. 4.**
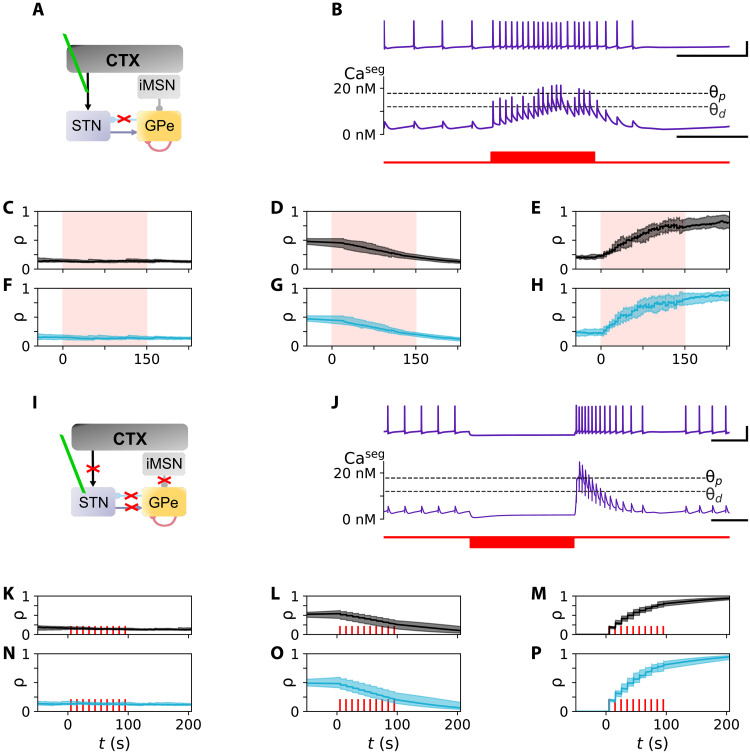
Reproducing plasticity experiments. (**A**) Model layout for simulating experiments by Chu *et al.* (*23*) where all non-NMDA–related synaptic currents onto the STN are blocked (red cross). Stimulation is applied at the CTX-to-STN connections (green line). (**B**) STN activity (top), Ca concentration within a dendritic segment, *Ca*^seg^, (center), and cortical stimulation, implemented as a 300-ms-long cortical firing rate increase to 50 Hz every 5 s (bottom). Dashed lines mark the depression, Θ_d_, and potentiation thresholds, Θ_p_, of the synaptic plasticity model (*48*). (**C** to **H**) No effect [(C) and (F)], LTD [(D) and (G)], or LTP [(E) and (H)] can be observed at CTX-to-STN [(C) to (E)] and GPe-to-STN [(F) to (H)] synapses following 150 s of stimulation (red) as shown by unchanged, reduced, or increased synaptic efficacies, ρ, respectively. (**I**) Model layout for simulating experiments by Wang *et al.* (*50*). (**J**) STN membrane potential (top) and *Ca*^seg^ (center) during a rebound burst induced by a hyperpolarizing current pulse of −3.7 μA/cm^2^ (bottom). Black horizontal bars mark time intervals of 200 ms, and vertical bars mark a voltage difference of 50 mV. (**K** to **P**) Repeated rebound bursts induced by a sequence of 10 current pulses (red bars) delivered to the STN at 0.1 Hz may lead to no change [(K) and (N)], LTD [(L) and (O)], or LTP [(M) and (P)] of CTX-to-STN [(K) to (M)] and GPe-to-STN [(N) to (P)] synapses. [(C) to (H)] and [(K) to (P)]: The median and interquartile range of results from 10 simulations are shown. Random initial synaptic efficacies with (mean, SD) = (0.33, 0.033) [(C), (F), (K), and (N)] or (0.5, 0.05) [(D), (G), (L), and (O)] were used. In (C), (F), (K), and (N), cortical inputs were as in the healthy state (Table 2). For LTD simulations either 300 ms of cortical bursting at a frequency of 8 Hz was applied every 5 s [(D) and (G)] or hyperpolarizing current pulses of −3.15 μA/cm^2^ [(L) and (O)]. See Materials and Methods and description of (B) and (J) for (E), (H), (M), and (P).

Heterosynaptic plasticity was modeled by specifying that each neuron has three dendritic segments, each containing local calcium concentrations that influence the strengths of all synapses in that segment (Fig. 3). To test whether our model can reproduce the dynamics of cortically evoked synaptic plasticity in the STN, we established an experimental setup similar to that of Chu *et al.* (*23*); cortical stimulation was delivered, and all synaptic inputs were blocked, except for CTX-to-STN NMDA receptors (see Fig. 4A). This protocol was capable of inducing either no synaptic change, long-term depression (LTD), or LTP of STN-bound synapses in our model. A representative STN neuron’s response to corresponding cortical input is shown in Fig. 4B. Calcium influx occurs through NMDA receptors when the time delay between pre- and postsynaptic spiking is short, increasing the local calcium concentrations in the dendritic segments (Fig. 4B). As both excitatory and inhibitory synapses may be bound to the same dendritic segment (*54*), acute changes in local calcium concentrations lead to copotentiation of both synapse types. Repeated exposure to cortical stimulation resulted in acute changes in STN calcium concentrations. This led to LTP in CTX-to-STN and GPe-to-STN synapses (Fig. 4, E and H), reproducing the effect of heterosynaptic plasticity (*23*). The strength of cortical inputs affects the shape of transient STN calcium concentration changes, and our model predicts that either no synaptic change (Fig. 4, C and F) or LTD (Fig. 4, D and G) can be observed for weaker cortical stimulation.

### Rebound bursting–induced long-term plasticity

In their experiments, Wang *et al.* (*50*) found that the strength of GPe-to-STN synapses could be altered via rebound bursting of STN neurons induced by hyperpolarizing current pulses. The number of spikes in a rebound burst determined whether no synaptic changes, LTD, or LTP occurred. They linked these effects to the quantity of calcium influx by systematically blocking T- and L-type calcium channels. Thus, their work established a direct link between STN synaptic plasticity and calcium dynamics.

To demonstrate that our computational model can reproduce the dynamics of inhibition-induced long-term plasticity, we established an experimental setup similar to theirs (see Fig. 4I) (*50*). Synaptic transmission was blocked (an isolated STN neuron was simulated), and hyperpolarizing current pulses were delivered.

A representative response of an STN neuron to a single current pulse is shown in Fig. 4J. Hyperpolarization deinactivates T-type calcium channels, facilitating calcium influx during the rebound burst (Fig. 4J) (*55*). We delivered hyperpolarizing current pulses of different strengths to induce rebound bursts with different numbers of spikes. As in Wang *et al.* (*50*), rebound bursts with only a few spikes did not change synaptic efficacy as calcium remained below the depression threshold (Fig. 4, K and N). Rebound bursts containing approximately four to eight spikes resulted in LTD as this threshold was crossed (Fig. 4, L and O). Rebound bursts with greater than eight spikes resulted in potentiation as calcium crossed the potentiation threshold (Fig. 4, M and P) (see fig. S2 for a more detailed analysis). Repeated exposure to rebound bursts with few or sufficiently large numbers of spikes led to corresponding changes of GPe-to-STN synapses (Fig. 4, N to P), similar to the results of Wang *et al.* (*50*).

These results support the hypothesis that homo- and heterosynaptic plasticity in the STN can be explained by a calcium-based synaptic plasticity mechanism similar to the one presented by Graupner and Brunel (*48*). Because of the lack of experimental evidence, we assumed that a similar plasticity model applies to the GPe, as heterosynaptic plasticity is seen in many diverse types of neurons (*51*). Specifically, we assumed that the same mechanism is present at all synapses except for the iMSN to GPe synapses (*26*, *56*).

### Increased indirect pathway activity triggers homeostatic synaptic reorganization in the STN-GPe circuit

In PD, the loss of striatal dopamine promotes hyperactivity of iMSNs. As explained by the direct/indirect pathway model, this leads to an imbalance between these two pathways, ultimately resulting in PD motor symptoms (*4*–*6*). As hypothesized by previous studies, compensatory synaptic reorganization in the BG might occur to counteract elevated iMSN activity (*22*). To test this hypothesis, we studied synaptic reorganization in response to a step-like transition to hyperactive iMSNs, implemented by replacing iMSNs with healthy activity by fast-spiking iMSNs (Fig. 2).

Our computational model incorporated homeostatic, calcium-based, activity-dependent structural plasticity based on the linear Butz and Van Ooyen algorithm (*49*, *57*). With this mechanism, the number of excitatory and inhibitory connections at each neuron changes in response to deviations of low pass–filtered somatic calcium concentrations from a target calcium concentration. Specifically, a surge in the low pass–filtered somatic calcium concentration will trigger an increase in the number of inhibitory and a decrease in the number of excitatory synapses. The timescale at which synaptic connections change is of the order of hours. Updates to synaptic connectivity occur every 200 ms. Each excitatory and inhibitory connection is associated with a randomly chosen segment on the postsynaptic neuron.

**Fig. 5.**
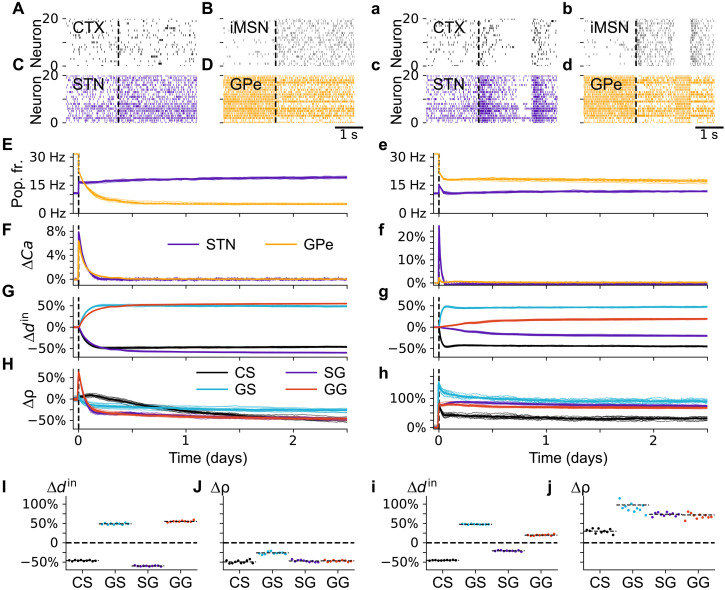
Step-like changes of iMSN and cortical inputs induce synaptic reorganization. (**A** to **J**) Step-like transition to hyperactive iMSNs at *t* = 0 hours, with high iMSN firing rate of 10 Hz while the cortical activity was kept unchanged [vertical dashed black lines in (A) to (H)]. [(A) to (D)] Raster plots before and after step-like iMSN change. For each curve in (E) to (H), thin lines correspond to traces from individual simulations, while thick lines correspond to the mean of 10 simulations. (E) Mean instantaneous firing rates in the STN and GPe throughout the simulation. (F) Change of population-averaged low pass–filtered somatic calcium concentration in STN and GPe relative to its value in the healthy state before the step-like change (pre-iMSN). (G) Change of average in-degree of different synapse types relative to in-degree pre-iMSN. (H) Average synaptic efficacies of each synapse type relative to pre-iMSN values. Final values are shown in (I) (average in-degrees) and (J) (average synaptic efficacies). Dots mark results for individual network realizations and horizontal black dashed lines represent percentage changes of averages (see Materials and Methods for details). We used the abbreviations CS for CTX-to-STN synapses, GS for GPe-to-STN synapses, SG for STN-to-GPe synapses, and GG for GPe-to-GPe synapses. (**a** to **j**) The same as (A) to (J), but cortical and iMSN inputs were step-like switched to low-frequency bursting (0.5 Hz) at *t* = 0 in which high activity phases are separated by phases of quiescence. The overall mean instantaneous firing rate of cortical and iMSNs after the activity change was 3.2 Hz (a) and 10 Hz (b), respectively (see Materials and Methods for details on bursting activity).

We first simulated networks for 13 hours to obtain stationary states. Ten stationary states were obtained from simulations beginning with random initial conditions for neuron and synapse gating variables and random connections between neurons (Materials and Methods). The spiking activity of STN and GPe neurons in the stationary state is illustrated in fig. S1. Structural plasticity led to the formation of highly selective GPe → STN connectivity formed with trial-averaged in-degrees of ≈9.7 corresponding to STN neurons typically receiving input from no more than 1.3% of GPe neurons. Estimates of corresponding values in rats are 2% (*58*). We performed simulations from each of these states, where cortical activity remained unchanged (Fig. 5A) and hyperactive iMSNs were modeled by Poisson spike generators with a constant firing rate of 10.0 Hz (Fig. 5B; see also Fig. 2); we recorded firing rates and calcium concentrations of each neuron, synaptic efficacies, and in-degrees of each synapse type. In the following text, we refer to the mean of these data.

The firing rate of STN neurons increased, and the firing rate of GPe neurons decreased in response to increased iMSN activity (Fig. 5, C, D, and E). This further caused transient elevations in somatic calcium concentrations in both the STN and GPe (Fig. 5F). In both nuclei, this increase was caused by elevated STN activity: inside the STN through voltage-gated calcium channels and inside the GPe through elevated calcium input from NMDA receptors at STN-GPe projections.

Synaptic reorganization was triggered as a result, including changes in the mean number of incoming connections to a neuron (in-degrees) (structural plasticity; Fig. 5G) and changes in synaptic efficacies (synaptic plasticity; Fig. 5H). Briefly, the former changes occur if the low pass–filtered calcium concentration deviates from a reference value (Materials and Methods), whereas the latter may occur if calcium concentrations within synaptic segments surpass threshold concentrations (Fig. 4), e.g., due to spike patterns including short periods of elevated (pre- or postsynaptic) spiking activity. We observed transient synaptic dynamics on the timescale, between several hours and 1.5 days, after which synaptic connectivity stabilized.

All synaptic efficacies were transiently enhanced but declined below their initial value by the end of the simulation (Fig. 5H). In addition to weakening individual synapses, structural plasticity promoted lower in-degrees for CTX-to-STN connections and STN-to-GPe connections and higher in-degrees for GPe-to-STN connections and GPe-to-GPe connections, corresponding to losses and gains of respective synapse types (Fig. 5G). These results support the hypothesis that elevated iMSN activity, which is at the core of the indirect/pathway model, triggers synaptic reorganization in the indirect pathway via synaptic and structural plasticity.

### Low-frequency bursts strengthen synaptic connections

We have shown that the iMSN hyperactivity is sufficient to alter synaptic connectivity in the STN-GPe circuit, even in the absence of iMSN bursting. However, in PD and related animal models, not only the firing rate of striatal iMSNs is elevated but CTX neurons and iMSNs often display correlated low-frequency bursting (*53*, *59*). Next, we tested how low-frequency burst inputs affect synaptic connectivity. Ten simulations were performed after establishing stationary states, as described above; we changed the activity of cortical and iMSN Poisson spike generators to a low-frequency burst pattern in a step-like manner while keeping the same mean firing rates as in the previous section, resembling experimentally observed bursting activity in PD (*53*, *59*).

As shown in Fig. 5 (c and e), the mean firing rate of STN neurons increased slightly, and that of GPe neurons decreased (Fig. 5d) following the cortical and iMSN activity change. However, GPe firing rates were higher than after a step-like transition to hyperactive iMSNs (compare Fig. 5, E and e). The transient change in the population-averaged low pass–filtered somatic calcium concentration in the STN shown in Fig. 5f was noticeably larger than in the previous section (Fig. 5F). In contrast, the change in GPe calcium concentration was lower (compare Fig. 5, e and E). Changes in in-degrees were qualitatively similar to those observed following the transition to hyperactive iMSNs (compare Fig. 5, i and I); however, the changes in in-degrees of STN-to-GPe connections and GPe-to-GPe connections (Fig. 5g) were weaker and occurred on a slower timescale. Larger calcium concentrations lead to more rapid structural changes (Materials and Methods), which caused more rapid adaptation in the STN than in the GPe (Fig. 5). In contrast to the hyperactive iMSNs, cortical and iMSN low-frequency bursting resulted in persistent increases in synaptic efficacies in the STN-GPe circuit (Fig. 5h).

Our computational results suggest that both scenarios—hyperactive iMSNs and low-frequency bursting—(with the same mean firing rate of iMSNs) led to qualitatively similar changes in the number of synapses (structural plasticity). In contrast, while a transition to hyperactive iMSNs resulted in weaker synapses in the STN-GPe circuit (Fig. 5J), cortical and iMSN low-frequency bursting, similar to that observed experimentally (*53*, *59*), led to stronger synapses (synaptic plasticity) (Fig. 5j). These results suggest distinct roles of synaptic and structural plasticity in shaping network adaptation in response to altered BG inputs.

### Synaptic plasticity shapes the evolution of beta oscillations in the STN-GPe circuit

**Fig. 6.**
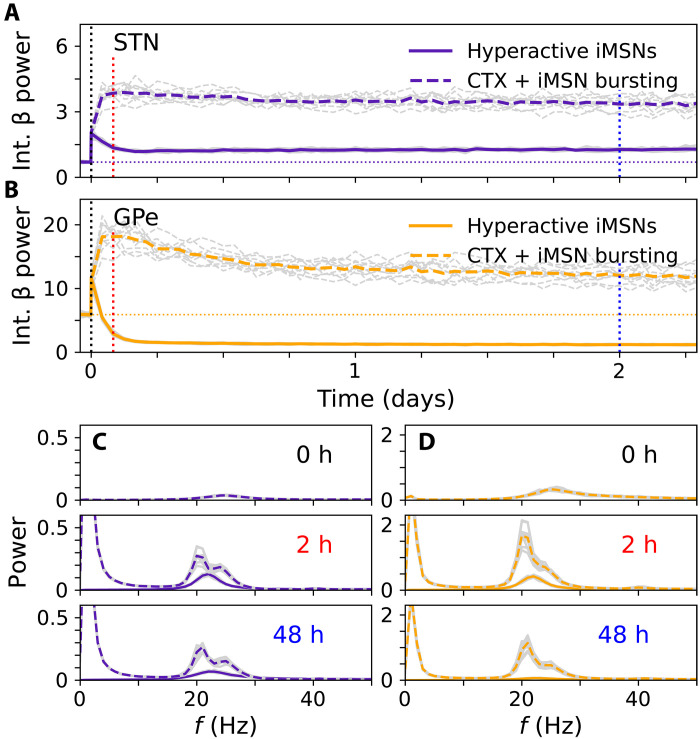
Evolution of beta power following step-like changes of input activity. Ten trials were simulated, including different random initial conditions and noise realizations. Colored lines mark averages over trials, and gray curves (**A** to **D**) show the results for individual trials. (A) Computational results showing the integral STN β power (integral power over a frequency range of 13 to 30Hz) for simulations of network response to step-like transition to hyperactive iMSNs (solid curves) and step-like change of CTX-iMSN burst input (dashed curves). The horizontal dotted line marks pre-input activity change conditions. (C) Corresponding STN power spectra at different times after activity change [labels and accordingly colored vertical lines in (A)]. [(B) and (D)] The same as (A) and (C) but for GPe activity. h, hours.

While the presence of beta oscillations per se is not pathological, and some amount of beta power is present in the healthy state (*17*, *18*), exaggerated beta oscillations are associated with akinetic motor symptoms (such as bradykinesia and rigidity) observed in patients with PD (*60*). Computational models suggest that alterations in neural connectivity can alter the level of synchronous beta oscillations (*32*, *61*). Therefore, we examined the time course and beta oscillation activity level in the STN and GPe populations following changes in iMSN and/or cortical activity discussed in the previous sections (Fig. 6). The following data were derived from 10 simulations, which continued from the stationary states established in our previous numerical experiments.

Both a transition to hyperactive iMSNs and to low-frequency bursting led to a rapid increase in STN and GPe beta power, which lasted several hours. Afterward, beta power stabilized at higher values than before the change, except for the GPe power, which stabilized at lower values after the transition to hyperactive iMSNs. Note that a transition to low-frequency bursting is necessary to observe increases in STN and GPe beta power, relative to the healthy state, that are comparable to the increases in STN and GPe LFP beta power observed in rats (*20*, *62*).

Membrane potentials of STN and GPe neurons in the presence of hyperactive iMSNs and low-frequency burst input are shown in the fig. S8. Traces of the average membrane potential suggest that beta oscillations are most pronounced at the beginning of iMSN and CTX low-frequency bursts. In our computational model, it took about 12 hours to a day for the beta power to stabilize.

### Differential impact of synaptic and structural plasticity

**Fig. 7.**
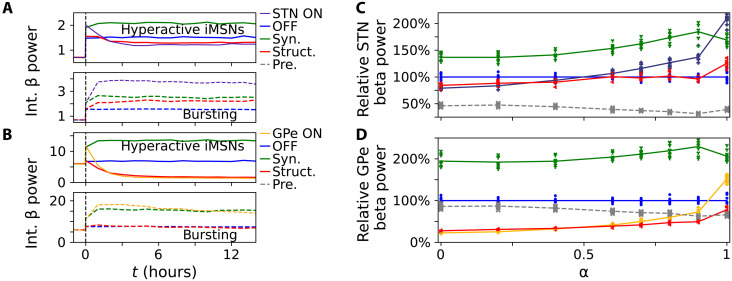
Effects of synaptic, structural, and no plasticity on beta power following step-like changes of input activity. Ten trials were simulated, including different random initial conditions and noise realizations. Curves mark averages over trials (**A** to **D**) and symbols results from individual trials [(C) and (D)]. [(A) and (B)] Comparison of trial-averaged integral STN (A) and GPe (B) beta power for simulations with synaptic and structural plasticity (ON), to simulations without plasticity (OFF), and to simulations in which either only synaptic plasticity was switched on (Syn.) or only structural plasticity was switched on (Struct.) after input activity change. [(C) and (D)] STN and GPe integral beta power *7* hours after step-like input activity change with different combinations of synaptic and structural plasticity relative to power in plasticity OFF condition for different degrees of iMSN and CTX bursting as specified by the parameter α (Materials and Methods). Dashed curve marks power pre activity change relative to OFF condition.

Next, we explored the contributions of both synaptic and structural plasticity to the emergence of beta oscillations following sudden changes in iMSN and/or cortical inputs. We performed simulations in which either synaptic plasticity, structural plasticity, or both types of plasticity were switched off. Corresponding results are shown in Fig. 7 (A and B). Following a sudden transition to hyperactive iMSNs, STN and GPe beta power increased even in the absence of any plasticity, but simulations with only synaptic plasticity displayed the highest beta power. Simulations with structural plasticity led to lower beta power, with GPe beta power even lower than before sudden input activity change. Following a sudden change to low-frequency burst input, beta power also increased even without plasticity. While simulations with synaptic plasticity displayed higher beta power, simulations with only structural plasticity led to a weaker increase in beta power than synaptic plasticity only. These results suggest that synaptic plasticity supports the emergence of beta oscillations, whereas the contribution of structural plasticity to the emergence of exaggerated beta oscillations depends on the iMSN and cortical inputs after the sudden change.

Next, we systematically varied the degree of bursting in the iMSN and cortical inputs after the sudden activity change. Specifically, we introduce the parameter α to scale the degree of bursting in iMSN and cortical inputs and to interpolate between the case of hyperactive iMSNs (α = 0) and low-frequency bursting (α = 1). Simulations with only synaptic plasticity displayed higher beta power than those without plasticity for all considered degrees of bursting (Fig. 7, C and D). In contrast, simulations with structural plasticity showed lower beta power for low degrees of bursting but similar, or even higher, beta power for high degrees of bursting (Fig. 7, C and D).

These results suggest that synaptic plasticity, in response to changes in iMSN and/or cortical input activity, increases STN and GPe beta power. In contrast, slower synaptic reorganization due to structural plasticity acts in a compensatory manner, counteracting elevated beta oscillations in the GPe and STN for low degrees of correlated low-frequency bursting. However, it becomes maladaptive in the sense that it increases beta power if too much bursting activity is present in cortical and iMSN inputs. Given the elevation in beta power observed in the absence of synaptic reorganization (Figs. 6C and 7), our results also suggest that a substantial portion of the beta power change is due to upstream changes of neuronal input activity in the indirect and/or hyperdirect pathway.

## DISCUSSION

### Synaptic reorganization as a compensatory mechanism

PD is accompanied by widespread activity changes and synaptic reorganization in the BG. Historically, models of BG dysfunction have suggested that increased iMSN activity (*4*, *5*), and/or exaggerated beta oscillations, contribute to akinetic PD motor symptoms such as rigidity and bradykinesia (*60*). However, the ways in which BG activity patterns and synaptic reorganization relate to each other and, ultimately contribute to PD motor symptoms are still in the process of being understood.

Previous studies suggested that some synaptic reorganization occurs as a compensatory mechanism to counteract neuronal activity changes in the indirect pathway that follow iMSN hyperactivity, in particular, the increase in the number and strength of GP to STN synapses and the depression of cortical to STN inputs (*22*, *32*). Motivated by this hypothesis, we explored the role of different plasticity mechanisms in synaptic reorganization following iMSN hyperactivity. To this end, we built a detailed computational model of long-term synaptic and structural plasticity in the STN-GPe circuit, a major part of the indirect pathway.

We incorporated the calcium-based synaptic plasticity mechanism presented by Graupner and Brunel (*48*) into a network model of the STN-GPe circuit, which contains conductance-based neuron and synapse models. In the model, two thresholds separate calcium concentrations for which either no synaptic change, synaptic depression, or synaptic potentiation occurs. Considering calcium concentrations inside individual synaptic segments, we showed that such calcium-based synaptic plasticity reproduces previous experimental results on hetero- and monosynaptic plasticity in rodent STN (*23*, *50*).

In our computational model, repeated bouts of elevated cortical inputs induced STN bursting and led to heterosynaptic plasticity of CTX-to-STN synapses and GPe-to-STN synapses (Fig. 4). NMDA receptor–mediated increases in STN segment calcium concentrations caused the latter. This agrees with previous experiments on heterosynaptic plasticity in mice models, namely, in which LTP of CTX-to-STN synapses and GPe-to-STN synapses was observed when presynaptic cortical axons and postsynaptic STN neurons were activated simultaneously (*23*). In experimental rat models, Wang *et al.* (*50*) studied monosynaptic plasticity in the STN, linking the strength of hyperpolarization-induced rebound bursting to changes in the strength of GPe-to-STN connections. Very weak rebound bursts induced minor to no changes in synaptic strength, weak bursts caused LTD, and strong bursts induced LTP (*50*). Simulating their experiments in rat brain slices in our computational model, we found that corresponding synaptic long-term changes occurred if rebound bursting–induced calcium changes led to no threshold crossing, crossing of the lower (depression) threshold, and crossing of the higher (potentiation) threshold, respectively (Fig. 4); however, the exact transition between depression and potentiation occurred at stronger bursts than in Wang *et al.*’s experiments (fig. S2) (*50*). The amount of Ca influx via NMDA receptors determines the strength of such calcium changes in excitatory synapses, whereas it is determined by the level of hyperpolarization (which, in turn, deinactivates T-type calcium channels) in inhibitory synapses. Through this mechanism, rebound bursting is critically linked to synaptic reorganization in the STN-GPe circuit.

In our model, we assumed that the synaptic plasticity mechanism proposed by Graupner and Brunel (*48*) applies to all synapses within the STN-GPe circuit, except for iMSN to GPe synapses. Further experiments in the GPe—similar to those published by Chu *et al.* (*23*) and Wang *et al.* (*50*)—could help to assess this. This is particularly interesting, as intra-GPe transmission is hypothesized to decorrelate GPe neuronal activity (*22*), thus suggesting that LTP in the GPe might counteract abnormal synchrony. Previous studies using a small-scale computational model of the STN-GPe network have found that spike timing–dependent plasticity of GPe-to-GPe connections alone can lead to the coexistence of synchronized and desynchronized states within this circuit (*63*).

### Modeling structural plasticity

In addition to calcium-based synaptic plasticity, we considered calcium-based structural plasticity by implementing a calcium-dependent structural plasticity rule similar to the Butz and Van Ooyen framework (*49*). This structural plasticity mechanism adds or removes synaptic connections based on the difference between the present calcium concentration and a target value, which, in turn, governs firing rates in the stationary state. Detailed knowledge about structural plasticity in the STN-GPe circuit is currently not available; however, widespread synaptic reorganization—in the form of changes in the density of synaptic boutons in the STN-GPe circuit—has been observed in animal models of PD in response to chronic DD (*24*, *25*, *27*, *28*, *64*). A loss of CTX-to-STN connections after chronic chemogenetic elevation of striato-pallidal transmission in mice in vivo has also been observed (*24*).

### Model predictions

Our computational model had several important predictions that are listed below.

1) LTP of CTX-to-STN synapses occurs after the repetitive delivery of strongly hyperpolarizing inhibitory current pulses to the STN.

2) Bursting input to the GPe via the STN, or GPe rebound bursting, may lead to LTP in the GPe (under the assumption that similar plasticity mechanisms also act in the GPe).

3) The strengths of CTX → STN and STN → GPe connections in the DD state vary depending on the degree of bursting in cortico-subthalamic and striato-pallidal inputs. Thus, the relative changes of these connections to control conditions may depend on the activity state.

4) A persistent increase of striatal input to GPe, as described by the direct/indirect pathway model, causes widespread structural changes in the STN-GPe circuit (i.e., a loss of CTX → STN and STN → GPe connections, and a gain of GPe → GPe and GPe → STN connections).

5) Exaggerated beta oscillations in the GPe require patterned iMSN activity, such as bursting. High firing rates of Poisson spiking in iMSNs alone are not sufficient.

6) In the dopamine-depleted state, our computational results suggest an increase in the number of intra-GPe connections and an increase in strengths of these synapses in the presence of bursting input from striatal and cortical inputs.

7) Synaptic plasticity in the STN-GPe circuit contributes to exaggerated beta oscillations in PD.

8) Structural plasticity counteracts exaggerated beta oscillations for low degrees of bursting in inputs to the STN-GPe circuit.

9) GPe beta may vary between activity states with different levels of bursting input to the STN-GPe circuit.

### Synaptic reorganization in response to DD

The observation of synaptic reorganization in the BG in response to chronic DD in animal models of PD prompted the discussion of how synaptic reorganization relates to altered neuronal activity in the DD BG (*22*). A critical question in this context is whether synaptic reorganization is the cause, or a consequence, of BG network dysfunction (*22*). To address this question, we reproduced elevated iMSN activity leading to the dominance of the akinetic indirect pathway, a key feature of BG dysfunction according to the classical direct/indirect pathway model (*4*–*6*). Then, we tested whether this would trigger synaptic reorganization and which plasticity mechanisms may specifically contribute to this synaptic reorganization. In our computational model, the iMSN firing rate increase triggered a series of synaptic changes throughout the STN-GPe circuit construct (Fig. 5). Such changes occurred both in the absence and presence of bursting of iMSN neurons. Most of these changes have previously been observed experimentally. Specifically, the loss of CTX-to-STN connectivity was observed in response to chemogenetically elevated striatal activity (*24*). After chronic DD in animal models of PD, experimental studies also found a loss of CTX-to-STN connectivity (*24*, *28*, *64*), a gain of GPe-to-STN synapses (*25*), and a loss of STN-to-GPe connectivity (*27*). Our results suggest that synaptic reorganization in the STN-GPe circuit is a consequence of altered striatal (and cortical) inputs. In the future, we plan on testing the “cause hypothesis,” i.e., whether changes in synaptic connectivity lead to changes of neuronal activities in the different nuclei.

Our computational model also provides insight into the potential mechanisms underlying synaptic reorganization in the STN-GPe circuit. In our simulations, elevated iMSN activity increased STN firing rates by suppressing inhibitory GPe inputs. Corresponding changes in STN and GPe firing rates after striatal DD have been observed experimentally (*20*, *22*, *62*). Firing rate changes were followed by an increase in the STN calcium concentrations because calcium influx through NMDA receptors is related to the product of pre- and postsynaptic firing rates (*65*). Elevations in the STN calcium concentrations were counteracted by structural plasticity; this caused a loss of excitatory CTX-to-STN connections and a gain of inhibitory GPe-to-STN connections (Fig. 5G). Despite inhibition by elevated iMSN activity, the GPe calcium concentrations increased because of the elevation in the (presynaptic) STN firing rate. The increase was counteracted by structural plasticity by a loss of STN-to-GPe connections and a gain of GPe-to-GPe connections.

### Challenging the existing structural plasticity paradigms

The loss of STN-to-GPe connections observed in this study [Figs. 5, I and i, and in 6-OHDA mice (*27*)], is difficult to reconcile with the traditional view that structural plasticity aims for homeostasis of neuronal activity, with calcium concentrations acting as a proxy signal for neural firing rates (*41*, *49*). With this traditional view, homeostatic structural plasticity should cause a gain of STN-to-GPe connections and a loss of GPe-to-GPe connections to counteract the GPe inhibition by elevated iMSN activity. However, because of our model’s calcium-based structural plasticity mechanism, the mean GPe calcium concentrations increased because of the elevation in presynaptic STN activity; this subsequently increased the opening time of postsynaptic NMDA receptors, facilitating calcium influx. This homeostatic mechanism maintains the mean GPe calcium concentrations and results in a loss of STN-to-GPe connections [as observed experimentally (*27*)] and a gain of GPe-to-GPe connections, synaptic changes that would be considered antihomeostatic in the traditional view where structural plasticity is set to balance firing rate deviations from a target value. The gain of GPe-to-GPe connections has not yet been observed experimentally to date; however, we propose that the increased transmission strength of GPe-to-GPe connections in 6-OHDA–lesioned rats (*26*) may be mediated by more GPe-to-GPe synapses and/or a change in the efficacy of existing synapses. Experimental results by Miguelez *et al.* (*26*), showed an increase in intra-GPe synaptic strengths by measuring amplitudes of miniature inhibitory postsynaptic currents in rat brain slices; however, no increase in their frequency, which would suggest an increase in the number of synapses, was observed in the DD state.

### Other BG activity changes in response to DD

Besides firing rate modifications, research into animal models of PD revealed a wide range of activity changes in the DD BG. One is pronounced low-frequency bursting of cortical and striatal projection neurons, observed in rodent models of PD (*53*, *59*). To test whether synaptic reorganization also occurred in response to cortical and iMSN low-frequency bursting, we modulated cortical and iMSN inputs accordingly. Low-frequency bursting in the cortex and striatum reduced the change in GPe afferent connectivity compared to hyperactive iMSNs without bursting (compare Fig. 5, I and i). At approximately 25%, the loss of STN-to-GPe connectivity was closer to the experimentally observed values for GPe-bound connections in 6-OHDA–lesioned mice (*27*) than after the transition to hyperactive iMSNs. Changes in the number of STN afferents were similar after switching to low-frequency burst input (Fig. 5i) and transitioning to hyperactive iMSNs (Fig. 5I). In our computational model, synaptic efficacies were enhanced after switching to low-frequency bursting.

Our results suggest that synaptic reorganization in the STN-GPe network strongly depends on iMSN and CTX activity characteristics, which vary in different brain states (*20*, *21*). In our computational model, structural reorganization was robust concerning input activity changes, but the dynamics of synaptic efficacies varied on the basis of input activity: Synaptic efficacies were enhanced after switching to low-frequency bursting but were reduced after a transition to hyperactive iMSNs (compare Fig. 5, j and J). The experimental findings regarding changes in synaptic strengths are unclear (*24*, *25*), likely reflecting input correlations (compare Figs. 5, I and J and i and j). Synaptic efficacy changes can occur on a timescale of minutes (*66*), suggesting that the strengths of individual synapses may depend on the experimental conditions in which recordings are made.

### Exploring the relationship between beta oscillations and elevated iMSN activity

PD is associated with the emergence of exaggerated beta oscillations; however, it is still under debate how such oscillations relate to motor symptoms (*12*, *32*). In rat (*20*, *21*) and nonhuman primate models of PD (*19*), a late onset of beta oscillations following chronic DD has been observed. This supports the hypothesis that the onset of beta oscillations results from slow adaptation processes triggered by DD that increases iMSN activity (*6*, *22*). We tested this hypothesis in our computational model by simulating synaptic reorganization due to calcium-dependent synaptic and structural plasticity triggered by a transition to iMSN hyperactivity (a state caused by the presence of dopaminergic lesions) (*53*). In our model, this transition was instantaneous, which makes it easier to identify the contributions of synaptic and structural plasticity to changes of firing rates and beta power in the STN and GPe. For example, elevating the iMSN rate leads to an immediate decrease in the GPe firing rate, followed by a further decrease due to structural reorganization (Fig. 5E). The individual contribution of both of these factors would not be as clear if the iMSN firing rate were slowly titrated to its final value.

Although our model reproduced known synaptic reorganization (Fig. 5), we did not observe a delayed onset of STN and GPe beta oscillations after step-like iMSN rate increase both with and without bursting (Fig. 6). This discrepancy might result from slower progression of DD within the striatum in experimental studies (*19*). Furthermore, elevated oscillatory activity was recorded in rats after complete but not after partial 6-OHDA lesion (*67*). The increase in the firing rates of all iMSNs in our model might resemble the effects of DD of major parts of the striatum or the complete lesion case. Considering a slow transition to hyperactive iMSNs resulted in a slow onset of STN beta power and a slow change of iMSN and CTX activity to low-frequency bursting resulted in slow onsets of STN and GPe beta power (figs. S3 and S4). It also remains a possibility that upstream changes in synaptic organization, in the motor cortex or striatum, lead to the generation of larger amplitude beta oscillations, which then propagated to the STN and GPe. In general, we find substantial variability in the intensity of beta oscillations depending on cortical and iMSN activity characteristics. This reflects the variety of experimental findings across different animal models and patients with PD (*9*, *12*), in particular, that exaggerated beta oscillations have not been observed in 6-OHDA–lesioned mice (*9*) and that beta oscillations in 6-OHDA rats and MPTP monkeys occur at different frequencies (*12*), where the frequency in rats closer resembles that observed in patients with PD. It has been suggested that this variability may result from the involvement of different subcircuits in beta oscillation generation (*32*) and the dependence of abnormal beta oscillations on the brain states (*21*).

### Synaptic and structural plasticity produce different changes in beta power

While synaptic plasticity changes synaptic efficacies if calcium surpasses specific threshold concentrations, structural plasticity leads to the generation of new and the removal of old synaptic connections in response to deviations from a target concentration. Performing simulations of our computational model without synaptic and/or structural plasticity, we found distinct contributions of either type of plasticity to elevated beta power after input activity changes. Synaptic plasticity alone led to increased beta power minutes after changing input activity (Fig. 6F), likely caused by the rapid increase of synaptic efficacies during the first minutes of the simulations (Fig. 5, H and h). In contrast, considering only structural plasticity typically reduced beta power, except for STN beta power following the switch to cortical and iMSN low-frequency bursting (Fig. 6, C and F). Together, these results demonstrate the essential role of different calcium-based plasticity mechanisms in the generation and/or maintenance of exaggerated beta oscillations in the STN-GPe circuit. They also suggest that synaptic plasticity in response to correlated inputs may enhance pathological oscillations in the BG and contribute to the brain state dependence of such oscillations, as inputs vary according to the state of activity. For instance, in 6-OHDA–lesioned rats, beta oscillations were observed in an active state but not during slow-wave sleep states (*21*, *62*). Note that a sudden change of iMSN and/or CTX activity led to some degree of elevated beta power, even in the absence of plasticity, indicating that a shift in inputs alone can also lead to elevated beta power in the model (see also figs. S6 and S7). In addition, we observed a dependence of beta power before input activity changes on coupling strengths and bias current (figs. S8 and S9). Elevated striatal firing rates reduce autonomous GPe activity in favor of elevated beta oscillations due to alternating GPe bursts and STN rebound bursts. Beta oscillations are most pronounced at the beginning of low-frequency iMSN and CTX bursts (fig. S10).

### Limitations of the study

Our computational model captures several key aspects of long-term synaptic reorganization within the STN-GPe circuit that have not been previously simulated; however, several limitations exist. Firstly, using single-compartment conductance-based neurons balances biophysical detail and computational efficiency, enabling simulation durations of several days with detailed calcium dynamics (Figs. 5 and 6). Second, our model lacks spatial details of synaptic connectivity and local calcium concentrations through the dendrites, which is suggested to play a vital role in governing heterosynaptic plasticity (*51*). Nevertheless, structural plasticity led to highly selective STN → GPe and GPe → STN connectivity as observed in rats (*58*). Next, short-term plasticity was not incorporated; however, we acknowledge that this may affect spike patterns such as bursting (*68*). Because of a lack of experimental data, we considered a single-fixed point version of the homeostatic plasticity rule presented by Butz and Van Ooyen (*49*, *57*). Although some experimental evidence supports the existence of a low-calcium fixed point for structural plasticity in the rat cerebral cortex (*69*), calcium levels in our simulations never reached such low concentrations. Butz and Van Ooyen’s study considered the structural plasticity of excitatory synapses (*49*). We assumed that synaptic and structural plasticity are present at the excitatory CTX-to-STN connections and STN-to-GPe connections, as well as the inhibitory GPe-to-GPe connections and GPe-to-STN connections. This was motivated by the observation of structural changes after chronic DD. Specifically, a reduction in the number of STN-to-GPe synpases and CTX-to-STN synpases was observed (*24*, *27*, *28*) and an increase in the number of GPe-to-STN synpases per axon terminal (*25*). Structural plasticity at inhibitory synapses has also been observed in other brain regions, e.g., in hippocampal cultures (*70*). To date, experimental evidence for structural changes in GPe-to-GPe connections has not been reported (*26*). The de novo creation of connections between pairs of presynaptic and postsynaptic neurons may be supported by reports of increased numbers of pallidal neurons responding to movements of body parts after chronic DD (*71*). Besides structural plasticity, evidence for the presence of synaptic plasticity has been reported for CTX-to-STN synapses (*72*), GPe-STN synapses (*23*, *50*), and GPe-to-GPe synapses (*26*). We also note that the calcium-based plasticity model developed by Graupner and Brunel approximates the complex secondary messenger signaling system underlying LTP and LTD (*47*).

Our investigation was limited to the STN-GPe circuit; thus, additional network-wide factors were not considered, which may be the reason why our model did not show the delayed onset of beta oscillations, following chronic DD, observed in animal models (*19*, *21*). These include subpopulations of GPe neurons with distinct activity and connectivity patterns (*27*, *73*), potentially contributing to the only partial reproduction of intra-GPe connectivity changes observed in rat brain slices (*26*) as discussed above, striato-pallidal loops in which exaggerated beta activity may originate (*32*), and feedback from the entire cortico-BG circuit. Furthermore, we did not model the corticostriatal pathway and considered individual cortical and striatal spike generators as independent. However, we considered low-frequency bursting in these populations to be in-phase, which was motivated by very short latencies (≈10 ms) of cortically evoked striatal responses (*74*). We aim to include this feedback in future versions of the computational model to study its effect on network structure and the onset of exaggerated beta oscillations. In our model, input from other brain areas is modeled using cortical and striatal spike generators whose activity is adapted to resemble healthy cortical and striatal activity (*23*, *53*, *59*), as well as Parkinsonian states with different degrees of bursting inputs (*24*, *53*, *59*).

Our results suggest that homeostatic calcium–based plasticity in the BG may contribute to the observed synaptic reorganization that occurs following chronic DD (*24*–*28*, *64*). Similar synaptic reorganization was generated by our computational model in response to elevated iMSN activity, a core tenet of the classical direct/indirect pathway model of BG dysfunction in PD (*4*–*6*). The model reproduced the experimentally observed increases in beta power in both the STN and GPe, as well as synaptic reorganization, only when elevated iMSN activity was combined with correlated bursting in the CTX and iMSNs. This suggests that correlated bursting contributes to the amplification of beta oscillations characteristic of PD. We also found distinct and partly opposite contributions of synaptic and structural plasticity to the emergence of beta oscillations. Whereas increased synaptic strengths due to synaptic plasticity tended to enhance beta oscillations, structural reorganization in response to elevated iMSN activity counteracted beta oscillations, but weakly opposed beta oscillations or even amplified them in response to low-frequency bursting input. These results strongly support the hypothesis that synaptic reorganization due to structural plasticity occurs as a corrective mechanism intended to counteract the effects of elevated iMSN input due to striatal dopamine depletion; however, the corrective nature of structural reorganization fails if iMSN and cortical input contain high degrees of bursting. In contrast, our results suggest that synaptic plasticity allows for an adjustment of synaptic strengths in response to changes in the BG input spike patterns.

## MATERIALS AND METHODS

### Computational model of long-term plasticity in the STN-GPe circuit

We developed a computational model of long-term plasticity in the STN-GPe circuit consisting of 250 STN and 750 prototypic GPe neurons (*75*) simulated using modified conductance-based models (*45*, *46*). The STN received 2500 excitatory inputs from the cortex, and the GPe received 7500 inhibitory inputs from the striatum, with each of these inputs simulated using Poisson spike generators. Connections from the cortex to the STN and from STN to GPe are excitatory and produce AMPA and NMDA postsynaptic currents. Connections from the striatum to the GPe, from GPe to STN, and from GPe to GPe are inhibitory and produce a GABA postsynaptic current (Fig. 1). A calcium-based plasticity rule governs synaptic conductances (*48*). Heterosynaptic plasticity is modeled by specifying that each neuron has three dendritic segments, which have local calcium concentrations that influence the strengths of all synapses onto that segment (Fig. 3). Although a full multicompartmental representation would more accurately capture the local diffusion of calcium within the dendritic tree, for computational efficiency, this process was approximated by a limited number of isolated dendritic segments. This approximation was necessary to simulate long-duration trajectories (see Fig. 5) required for the study of structural plasticity. Each excitatory and inhibitory connection is associated with a randomly chosen segment on the postsynaptic neuron. The number of excitatory and inhibitory connections received by each STN and GPe neuron is controlled by a homeostatic structural plasticity rule that responds to changes in somatic calcium concentrations on a timescale of hours (*49*). Synaptic and structural plasticity of MSN to GPe synapses are not simulated as it has been reported that no change occurs at the synapses between the iMSN and prototypic GPe neurons in 6-OHDA–lesioned rodents (*26*, *56*).

#### Conductance-based neuron models

Single-compartment, point neurons were used to model STN and GPe neurons (*45*, *46*). The dynamics of the membrane potential $V_{i}$ of neuron *i* is described by

$$C\frac{{\mathrm{dV}}_{i}}{\mathrm{dt}}=-I_{i}^{\text{ion}}+I_{i}^{\text{bias}}-\sum_{j_{i}}I_{{\mathrm{ij}}_{i}}^{\text{syn}}$$ (1)

where *C* is the membrane capacitance, $I_{i}^{\text{ion}}$ is the sum of all ionic currents, $I_{i}^{\text{bias}}$ is the applied bias current, and the last term is the sum over all synaptic input currents, where $j_{i}$ is the index of the $j_{i}$ th synapse of the postsynaptic neuron *i*. Following, the index *i* refers to quantities associated with the *i*th neuron.

The total ionic current for STN neurons is given by

$$I_{i}^{\text{ion}}=I_{i}^{\text{Na}}+I_{i}^{K}+I_{i}^{T}+I_{i}^{\text{Ca}-K}+I_{i}^{A}+I_{i}^{L}+I_{i}^{\text{leak}}$$ (2)

and for GPe neurons, it is given by

$$I_{i}^{\text{ion}}=I_{i}^{\text{Na}}+I_{i}^{K}+I_{i}^{T}+I_{i}^{\text{Ca}-K}+I_{i}^{\text{leak}}$$ (3)

More details on individual ionic currents are detailed in Table 1.

**Table 1.** STN and GPe ionic currents. Solid horizontal line separates STN-only currents (below) from STN and GPe ionic currents (above).

Symbol	Name	Equation
$I_{i}^{\text{Na}}$	Sodium current	$g^{\text{Na}}m_{i}^{3}h_{i}\left(V_{i}-E^{\text{Na}}\right)$
$I_{i}^{K}$	Kv3-type potassium current	$g^{K}n_{i}^{4}\left(V_{i}-E^{K}\right)$
$I_{i}^{T}$	T-type calcium channel	$g^{T}p_{i}^{2}q_{i}\left(V_{i}-E_{i}^{\text{Ca}}\right)$
$I_{i}^{\text{Ca}-K}$	Calcium-activated potassium current	$g^{\text{Ca}-K}r_{i}^{2}\left(V_{i}-E^{K}\right)$
$I_{i}^{\text{leak}}$	Leak current	$g^{\text{leak}}\left(V_{i}-E^{\text{leak}}\right)$
$I_{i}^{\text{A}}$	Voltage-dependent A-type potassium current	$g^{\text{A}}a_{i}^{2}\left(V_{i}-E^{\text{K}}\right)$
** $I_{i}^{\text{L}}$ **	**L-type long-lasting calcium current**	** $g^{\text{L}}c_{i}^{2}d_{1i}d_{2i}\left(V_{i}-E_{i}^{\text{Ca}}\right)$ **

Each ionic current is composed of a conductance $g^{X}$ , where *X* specifies the ionic current, a reversal potential $E^{X}$ , and one or more gating variables *x*. The conductances are given in table S3. The reversal potentials are constant for sodium ( $E^{\text{Na}}=40$ mV), potassium ( $E^{K}=-90$ mV), and leak ( $E^{\text{leak}}=-60$ mV) currents, whereas the reversal potential for calcium, $E^{\text{Ca}}$ , is derived from the ratio of the external calcium concentration, ${\left[{\text{Ca}}^{\text{soma}}\right]}^{\text{out}}$ , to the internal calcium concentration within the soma of neuron *i*, ${\left[{\text{Ca}}^{\text{soma}}\right]}_{i}^{\text{in}}$ , using the Nernst equation

$$E_{i}^{\text{Ca}}=\frac{\mathrm{RT}}{\mathrm{ZF}}\text{ln}\left(\frac{{\left[{\text{Ca}}^{\text{soma}}\right]}^{\text{out}}}{{\left[{\text{Ca}}^{\text{soma}}\right]}_{i}^{\text{in}}}\right)$$ (4)

where *R* is the universal gas constant, *T* = 303.15 K is the temperature, *Z* = 2 is the valence of the calcium ions, *F* is Faraday’s constant, and the external calcium concentration is set to ${\left[{\text{Ca}}^{\text{soma}}\right]}^{\text{out}}=2$ μM (*46*).

The gating kinetics are governed by the equation

$$\frac{{\mathrm{dx}}_{i}}{\mathrm{dt}}=\frac{x_{i}^{\infty }-x_{i}}{\tau_{i}^{x}}$$ (5)

where $x\in \left\{a,b,c,d_{1},d_{2},h,m,n,p,q,r\right\}$ corresponds to one of the gating variables listed in tables S1 and S2.

The steady state (in)activation functions, $x_{i}^{\infty }$ , are given by

$$x_{i}^{\infty }={\left[1+\text{exp}\left(\frac{M_{i}+\Theta^{x}}{k^{x}}\right)\right]}^{-1}$$ (6)

where $\Theta^{x}$ are the half (in)activation voltages and $k^{x}$ are the half (in)activation slopes. The membrane potential, $M_{i}=V_{i}$ , governs the response of all gates except $d_{2}$ and *r*, which are governed by the internal somatic calcium concentration $M_{i}={\left[{\text{Ca}}^{\text{soma}}\right]}_{i}^{\text{in}}$.

The fast (in)activation timescales, i.e., $x\in \left\{a,d_{2},m,r\right\}$ , are given by

$$\tau_{i}^{x}=\tau^{x,0}+\tau^{x,1}{\left[1+\text{exp}\left(-\frac{M_{i}+\Theta^{x,1}}{\sigma^{x,1}}\right)\right]}^{-1}$$ (7)

The slow (in)activation timescales, i.e., $x\in \left\{b,c,d_{1},h,n,p,q\right\}$ , are given by

$$\tau_{i}^{x}=\tau^{x,0}+\tau^{x,1}{\left[\text{exp}\left(\frac{M_{i}+\Theta^{x,1}}{\sigma^{x,1}}\right)+\text{exp}\left(\frac{M_{i}+\Theta^{x,2}}{\sigma^{x,2}}\right)\right]}^{-1}$$ (8)

The value for each gating variable may be found in tables S1 and S2.

#### Somatic and segmental calcium dynamics

The somatic calcium concentration, ${\left[{\text{Ca}}^{\text{soma}}\right]}_{i}^{\text{in}}$ , of a given neuron *i* governs the behavior of calcium-dependent currents via ligand gating and influences the structural plasticity as described below. The calcium concentration at segment $l_{i}$ on a neuron *i*, ${\left[{\text{Ca}}^{\text{seg}}\right]}_{l_{i}}^{\text{in}}$ , governs the strength of the synapses associated with that segment, via the mechanism described by Graupner and Brunel (*48*). The somatic calcium dynamics are described using the differential equation presented by Otsuka *et al.* (*46*), which we extended by an additional calcium current arising from the NMDA receptors

$$\frac{d{\left[{\text{Ca}}^{\text{soma}}\right]}_{i}^{\text{in}}}{\mathrm{dt}}=-\frac{I_{i}^{T}+I_{i}^{L}+\sum_{j_{i}}I_{j_{i}}^{\text{NMDA},\text{Ca}}}{\mathrm{ZF}}-k_{\text{Ca}}{\left[{\text{Ca}}^{\text{soma}}\right]}_{i}^{\text{in}}$$ (9)

where $k_{\text{Ca}}=2$ ms^−1^ is the calcium removal rate, $\sum_{j_{i}}I_{j_{i}}^{\text{NMDA},\text{Ca}}$ is the calcium influx through all NMDA receptors, where $j_{i}$ refers to the $j_{i}$ th synapse of neuron *i*. Similarly, the dynamics of the calcium concentrations in the three segments of neuron *i* are described by

$$\frac{d{\left[{\text{Ca}}^{\text{seg}}\right]}_{l_{i}}^{\text{in}}}{\mathrm{dt}}=-\frac{I_{i}^{T}+I_{i}^{L}+\sum_{j_{i}}1_{A}\left(j_{i},l_{i}\right)I_{j_{i}}^{\text{NMDA},\text{Ca}}}{\mathrm{ZF}}-k^{\text{Ca}}{\left[{\text{Ca}}^{\text{seg}}\right]}_{l_{i}}^{\text{in}}$$ (10)

$l_{i}=0,1,2$ refers to individual segments of neuron *i* (Fig. 7). The NMDA current will only contribute to the change in calcium concentration if it synapses onto segment $l_{i}$ , as denoted by the indicator function $1_{A}\left(j_{i},l_{i}\right)$ , which returns one if synapse $j_{i}$ connects to segment $l_{i}$ , and zero otherwise.

#### Synapses

Synaptic conductances were represented using the model of Rubin *et al.* (*47*). Ligand gating of currents at the synapse *j* is governed by rise, $s_{j}^{y,\text{rise}}$ , fast, $s_{j}^{y,\text{fast}}$ , and slow components, $s_{j}^{y,\text{slow}}$

$$\frac{{\mathrm{ds}}_{j}^{y,\text{rise}}}{\mathrm{dt}}=-\Phi^{y}\left(1-s_{j}^{y,\text{fast}}-s_{j}^{y,\text{slow}}\right)f^{\text{pre}}\left(t\right)-\frac{s_{j}^{y,\text{rise}}}{\tau^{\text{rise},y}}$$ (11)

$$\frac{{\mathrm{ds}}_{j}^{y,\text{fast}}}{\mathrm{dt}}=\Phi^{y}\left(v^{y,\text{fast}}-s_{j}^{y,\text{fast}}\right)f^{\text{pre}}\left(t\right)-\frac{s_{j}^{y,\text{fast}}}{\tau^{\text{fast},y}}$$ (12)

$$\frac{{\mathrm{ds}}_{j}^{y,\text{slow}}}{\mathrm{dt}}=\Phi^{y}\left(v^{y,\text{slow}}-s_{j}^{y,\text{slow}}\right)f^{\text{pre}}\left(t\right)-\frac{s_{j}^{y,\text{slow}}}{\tau^{\text{slow},y}}$$ (13)

Here, *y* specifies the type of neurotransmitter, i.e., GABA_a_, AMPA, or NMDA. $f^{\text{pre}}$ is a step pulse lasting 1 ms, modeling presynaptic transmitter release, which is turned on to activate the postsynaptic synapse, *j*, when a presynaptic spike occurs. Φ is the rate of increase of the gating variables when neurotransmitter is released. $v^{y,\text{slow}}$ and $v^{y,\text{fast}}$ are attractor values for the slow and fast gates, and $\tau^{\text{rise},y}$ , $\tau^{\text{fast},y}$ , and $\tau^{\text{slow},y}$ are the time constants of the rise, fast, and slow components, respectively. The value of each parameter involved in synaptic gating may be found in table S4.

The current from an inhibitory synapse, i.e., $y={\text{GABA}}_{a}$ , is given by

$$\begin{array}{c} I_{j_{i}}^{{\text{GABA}}_{a}}=\\ -g_{j_{i}}^{{\text{GABA}}_{a}}\left(s_{j_{i}}^{{\text{GABA}}_{a},\text{rise}}+s_{j_{i}}^{{\text{GABA}}_{a},\text{fast}}+s_{j_{i}}^{{\text{GABA}}_{a},\text{slow}}\right)\left(V_{i}-E^{{\text{GABA}}_{a}}\right) \end{array}$$ (14)

Here, the index $j_{i}$ refers to the $j_{i}$ th synapse of the postsynaptic neuron *i*. $g^{{\text{GABA}}_{a}}$ is the maximum conductance, $E^{{\text{GABA}}_{a}}=-80$ mV the synaptic reversal potential, and $V_{i}$ the membrane potential of the postsynaptic neuron *i*.

Similarly, the current from the fast AMPA channel of excitatory synapses, with *y* = AMPA, takes the following form

$$\begin{array}{c} I_{j_{i}}^{\text{AMPA}}=\\ -g_{j_{i}}^{\text{AMPA}}\left(s_{j_{i}}^{\text{AMPA},\text{rise}}+s_{j_{i}}^{\text{AMPA},\text{fast}}+s_{j_{i}}^{\text{AMPA},\text{slow}}\right)\left(V_{i}-E^{\text{AMPA}}\right) \end{array}$$ (15)

with $g^{\text{AMPA}}$ being the conductance, and $E^{\text{AMPA}}=0$ mV the reversal potential of the AMPA channels.

In addition, the excitatory synapse model contains NMDA receptors (*y* = NMDA). Current flow through the NMDA receptors is divided into two components, a calcium current, $I^{\text{NMDA},\text{Ca}}$ , and nonspecific cationic current, $I^{\text{NMDA},\text{syn}}$ (*47*)

$$\begin{array}{c} I_{j_{i}}^{\text{NMDA},z}=\\ -g_{j_{i}}^{\text{NMDA},z}m^{z}\left(s_{j_{i}}^{\text{NMDA},\text{rise}}+s_{j_{i}}^{\text{NMDA},\text{fast}}+s_{j_{i}}^{\text{NMDA},\text{slow}}\right)\left(V_{i}-E^{z,\text{rev}}\right) \end{array}$$ (16)

Here, z=Ca or z=syn . The effect of magnesium block removal on the opening of NMDA receptors is modeled by $m^{z}$ with (*47*)

$$m^{\text{NMDA},\text{Ca}}=\frac{1}{1+0.6e^{-0.124V_{i}}}$$ (17)

and

$$m^{\text{NMDA},\text{syn}}=\frac{1}{1+0.6e^{-0.062V_{i}}}$$ (18)

To reproduce the ratios of NMDA-to-AMPA currents observed in CTX-to-STN synapses (*23*) and STN-to-GPe synapses (*27*), the following relation was assumed $g^{\text{NMDA},\text{syn}}=1.7g^{\text{AMPA}}$ . This ensured that the NMDA:AMPA current ratio, recorded under depolarized and hyperpolarized conditions, respectively, was 0.25:1.

#### Synaptic plasticity

To incorporate synaptic plasticity, we described the individual fast excitatory and inhibitory conductances, $g\in \left\{g^{{\text{GABA}}_{a}},g^{\text{AMPA}}\right\}$ , by

$$g_{j}^{y}=w^{y,\text{min}}+\left(w^{y,\text{max}}-w^{y,\text{min}}\right)\rho_{j}^{y}$$ (19)

where $\rho_{j}^{y}\in \left[0,1\right]$ represents the synapse’s efficacy, and $w^{y,\text{min}}$ and $w^{y,\text{max}}$ are the minimum and maximum synaptic conductances of synapse type y, respectively. Following, we suppress the index *j* to avoid bulky terms. The dynamics of $\rho^{y}$ are described using the calcium-based plasticity model presented by Graupner and Brunel (*48*)

$$\begin{array}{l} \tau^{\rho }\frac{d\rho^{y}}{\mathrm{dt}}=-\rho^{y}\left(1-\rho^{y}\right)\left(0.5-\rho^{y}\right)+\gamma^{p}\left(1-\rho^{y}\right)H\left({\left[{\text{Ca}}^{\text{seg}}\right]}^{\text{in}}-\theta^{y,p}\right)\\ \;-\gamma^{d}\rho^{y}H\left({\left[{\text{Ca}}^{\text{seg}}\right]}^{\text{in}}-\theta^{y,d}\right)+\sigma \sqrt{\tau }H\left({\left[{\text{Ca}}^{\text{seg}}\right]}^{\text{in}}-\theta^{y,d}\right)\xi^{y}\left(t\right) \end{array}$$ (20)

where ${\left[{\text{Ca}}^{\text{seg}}\right]}^{\text{in}}$ is the calcium concentration in the postsynaptic segment. The timescale of calcium-based synaptic plasticity is given by $\tau^{\rho }=30$ s, which is in the range suggested by Graupner and Brunel (*48*). The cubic term on the right-hand side of Eq. 20, describes the dynamics of $\rho_{j}^{y}$ in the absence of calcium fluctuations. In that case, there are two stable fixed points at $\rho_{j}^{y}=0$ and $\rho_{j}^{y}=1$ corresponding to down and up states, respectively, and an unstable fixed point at $\rho_{j}^{y}=0.5$ . The second term describes synaptic potentiation, scaled by $\gamma^{p}$ . *H(x)* is the Heavyside step function that returns a value of one if x≥0 and a value of zero otherwise. Thus, the potentiation term contributes to the dynamics of $\rho_{j}^{y}$ if the segment’s calcium concentration ${\left[{\text{Ca}}^{\text{seg}}\right]}^{\text{in}}$ is greater than the potentiation threshold $\theta^{y,p}$ . The third term describes depotentiation of synapses when the segment’s calcium concentration is above the depression threshold $\theta^{y,d}$ . The final term describes activity-dependent noise. This noise is only present when the calcium concentration is above the depression threshold, $\theta^{y,d}$ . $\xi_{j}^{y}\left(t\right)$ is zero-mean white Gaussian noise, $\langle \xi_{j}^{y}\left(t\right)\rangle =0$ and $\langle \xi_{j}^{y_{1}}\left(t\right)\xi_{k}^{y_{2}}\left(t^{\prime }\right)\rangle =\delta \left(t-t^{\prime }\right)\delta_{j,k}\delta_{y_{1},y_{2}}$ . Here, δ(x) is the Dirac delta distribution and $\delta_{a,b}=1$ if *a* = *b* and zero otherwise.

#### Structural plasticity

We incorporated a model of homeostatic, calcium-based, activity-dependent structural plasticity based on the linear Butz and Van Ooyen algorithm (*49*, *57*) into the neurons of the STN and GPe. The original algorithm governed synaptic connections between excitatory neurons and was modified here to incorporate inhibitory synapses. Each neuron contains a number of axonal and dendritic elements, which can merge with their opposing element on another neuron to form synapses. Excitatory axonal elements may merge with available excitatory dendritic elements to form new excitatory synapses, and likewise available inhibitory axonal and dendritic elements may merge to form inhibitory synapses. The number of excitatory dendritic elements, $z_{i}^{\mathrm{exc}}$ , and inhibitory dendritic elements, $z_{i}^{\mathrm{inh}}$ , available within a given neuron evolves on the basis of the dynamics of the long-term average calcium concentration ${\text{Ca}}_{i}^{L}$ . The dynamics of ${\text{Ca}}_{i}^{L}$ are described as a simple low-pass filter of the somatic calcium concentration

$$\tau^{\text{Ca},L}\frac{d{\text{Ca}}_{i}^{L}}{\mathrm{dt}}=-{\text{Ca}}_{i}^{L}+{\left[{\text{Ca}}^{\text{soma}}\right]}_{i}^{\text{in}}$$ (21)

Here, $\tau^{\text{Ca},L}=10$ s is the slow timescale at which the calcium concentration is averaged. Unlike in the Butz and van Ooyen model, the numbers of excitatory, $a_{i}^{\text{exc}}$ , and inhibitory axonal elements, $a_{i}^{\text{inh}}$ , remain static at a large value $a_{i}^{\text{exc}}=a_{i}^{\text{inh}}=200$ . Thus, the formation of synapses depends only on the somatic calcium concentration in the postsynaptic neuron. The change in the number of available excitatory or inhibitory dendritic elements is a linear function of ${\text{Ca}}_{i}^{L}$ , given by

$$\tau^{z}\frac{{\mathrm{dz}}_{i}^{\text{exc}}}{\mathrm{dt}}=1-\frac{{\text{Ca}}_{i}^{L}}{{\text{Ca}}_{i}^{L*}}$$ (22)

and

$$\tau^{z}\frac{{\mathrm{dz}}_{i}^{\text{inh}}}{\mathrm{dt}}=-1+\frac{{\text{Ca}}_{i}^{L}}{{\text{Ca}}_{i}^{L*}}$$ (23)

respectively.

The time constant for element growth is set to $\tau^{z}=3600$ s such that structural reorganization in Fig. 5 occurred within 2 days after changes of iMSN inputs (*24*). In the supplement, we show that shorter $\tau^{z}$ lead to similar structural changes (fig. S5). ${\text{Ca}}_{i}^{L*}$ is the target value for ${\text{Ca}}_{i}^{L}$ . Homeostasis of ${\text{Ca}}_{i}^{L}$ occurs as a result of the relationships established in the above equations, such that when ${\text{Ca}}_{i}^{L}$ falls below ${\text{Ca}}_{i}^{L*}$ , excitatory connections are added and inhibitory connections are lost, and vice versa when ${\text{Ca}}_{i}^{L}$ is greater than ${\text{Ca}}_{i}^{L*}$ . The number of vacant excitatory $z_{i}^{\text{exc},\text{vac}}$ or inhibitory elements $z_{i}^{\text{inh},\text{vac}}$ is the floor of the difference between the number of available and the number of bound elements $z_{i}^{\text{exc},\text{bou}}$ , $z_{i}^{\text{inh},\text{bou}}$ , given by $z_{i}^{\text{exc},\text{vac}}=\left\lfloor z_{i}^{\text{exc}}-z_{i}^{\text{exc},\text{bou}}\right\rfloor$ , or $z_{i}^{\text{inh},\text{vac}}=\left\lfloor z_{i}^{\text{inh}}-z_{i}^{\text{inh},\text{bou}}\right\rfloor$ . Synapses may form when vacant axonal and dendritic elements are available. While the variables evolved continuously at each time step, the updates in connectivity were simulated to occur every 200 ms. At each connectivity update, a connection could be formed between a vacant dendritic element and a randomly chosen axonal element, with probability 0.1.

#### Cortical and iMSN inputs

The activity of individual cortical and iMSN afferents projecting to the STN and GPe, respectively, were modeled by Poisson spike generators. We considered different types of spike generators: spiking and pause-bursting ones. Spiking afferent inputs were modeled as Poisson spike trains, with constant rate parameter $\bar{f}$ . Pause-bursting activity was realized by switching between a high rate (bursting), a low rate (spiking), and no activity (pause).

Bursting occurred at an average frequency of $F^{\text{bo}}=1/d^{\text{bo}}$ , where the time between subsequent burst onsets, $t^{b}$ , was drawn from a normal distribution with mean $d^{\text{bo}}$ and an SD of 0.1 s. During bursts, $n^{b}$ spikes with exponentially distributed interspike intervals were generated with mean interval $1/f^{b}$ and Poisson-distributed $n^{b}$ with $\lambda =f^{b}d^{b}$ , where $d^{b}$ measures the burst duration. After the last spike, the afferent switched to spiking mode with rate parameter $f^{s}$ until $t^{b}-d^{p}$ with $f^{s}=\frac{\bar{f}d^{\text{ob}}-f^{b}d^{b}}{d^{\text{ob}}-d^{b}-d^{p}}$ , ensuring an overall mean firing rate of $\bar{f}$.

To model different input activity patterns, we varied the rate parameter for spiking afferents, $\bar{f}$ , as well as the portion of bursting afferents, $P^{b}$ , and the parameters characterizing their activity pattern. In particular, we considered a healthy state (*53*, *59*), as well as a state of hyperactive iMSNs [motivated by the direct/indirect pathway model (*4*, *5*) as well as persistent chemogenetic excitation of striatal neurons in (*24*)] and a PD burst state [motivated by pause-bursting observed in (*53*, *59*)]. Corresponding parameters are listed in Table 2. In Fig. 7, we further introduced the parameter α to interpolate between the two PD states such that for any parameter *x* in Table 2, its value in the PD state obtained by interpolation *x*(α) is given by $x\left(\alpha \right)=\left(1-\alpha \right)x^{\text{hyperactive iMSNs}}+\alpha x^{\text{PD burst}.}$ . Thus, for α = 0, we retrieve the hyperactive iMSNs, and for α = 1, we retrieve the PD burst state in Table 2.

**Table 2.** Activity parameters for spike generators under different conditions. Values were taken where appropriate from the available literature (*23*, *24*, *53*, *59*). Here, “hyperactive iMSNs” and “PD burst.” refer to PD states with elevated but fixed iMSN firing rates and time-dependent iMSN and cortical firing rates mimicking low-frequency bursting, respectively (see also Fig. 2).

Parameter		CTX value	MSN value	CTX ref.	MSN ref.
**Healthy**					
Mean firing rate	$\bar{f}$	3.2 Hz	2.1 Hz	Rats (*59*)	Rats (*53*)
Proportion of bursting spikers	$P^{b}$	0.2	0.7	Mice (*23*)	Rats (*53*)
Burst occurrence frequency	$F^{B}$	0.2 Hz	0.22 Hz	Mice (*23*)	Rats (*53*)
Burst duration	$d^{b}$	0.2 s	0.34 s	Mice (*23*)	Rats (*53*)
Pause duration	$d^{p}$	0.0 s	0.0 s	Rats (*59*)	Rats (*53*)
Intra-burst firing rate	$f^{b}$	50 Hz	20 Hz	Mice (*23*)	Rats (*53*)
**Hyperactive iMSNs**					
Mean firing rate	$\bar{f}$	3.2 Hz	10.0 Hz	Rats (*59*)	Mice (*24*)
Proportion of bursting spikers	$P^{b}$	0.2	0.0	Mice (*23*)	N/A
Burst occurrence frequency	$F^{B}$	0.2 Hz	0.0 Hz	Mice (*23*)	N/A
Burst duration	$d^{b}$	0.2 s	0.0 s	Mice (*23*)	N/A
Pause duration	$d^{p}$	0.0 s	0.0 s	Rats (*59*)	N/A
Intra-burst firing rate	$f^{b}$	50 Hz	0 Hz	Mice (*23*)	N/A
**PD burst.**					
Mean firing rate	$\bar{f}$	3.2 Hz	10.0 Hz	Rats (*59*)	Rats (*53*)
Proportion of bursting spikers	$P^{b}$	1	1	Rats (*59*)	Rats (*53*)
Burst occurrence frequency	$F^{B}$	0.5 Hz	0.5 Hz	Rats (*59*)	Rats (*53*)
Burst duration	$d^{b}$	0.5 s	0.26 s	Rats (*59*)	Rats (*53*)
Pause duration	$d^{p}$	0.6 s	0.6 s	Rats (*59*)	Rats (*53*)
Intra-burst firing rate	$f^{b}$	10 Hz	34 Hz	Rats (*59*)	Rats (*53*)

### Model fitting procedure

The model, in its default state, was tuned to reflect the dopamine-intact STN-GPe circuit of the rat, for which the neural firing patterns and rates are well-described in the literature (*52*, *53*). To reduce the computational cost associated with large simulations, the number of STN neurons was set to 250 and the number of GPe neurons was 750, in accordance with the ratio of approximately 1:3 reported in (*75*). The values of $w^{\left(\text{min}\right)}$ [and by extension $w^{\left(\text{max}\right)}$ ] were manually adjusted to meet a number of specific constraints. Following the approach described by Fountas and Shanahan (*76*) for tuning the firing rates of STN and GPe neural populations, STN mean firing rates were first constrained to 10 ± 1 Hz (*52*, *77*), and GPe mean firing rates to 30 ± 1 Hz (*53*, *78*) to reflect activity in the dopamine-intact state in the rat. In addition to maintaining target firing rates, the strength of the GPe-to-STN connections was also tuned so that correlated firing in the GPe could induce rebound bursts in the STN. Specifically, the GPe-to-STN weight was adjusted so that coincident firing from 10 presynaptic GPe neurons should be sufficient to hyperpolarize the STN to < −75 mV, ensuring that the GPe was capable of deinactivating T-type calcium channels in the STN (*55*). MSN-to-GPe connections were tuned to values that ensured that MSN activity above approximately 30 Hz (the intra-burst firing frequency in 6-OHDA–lesioned MSN) could transiently silence the GPe (*53*). Last, as intrinsically generated beta power should be relatively low in the default state, synaptic weights were adjusted such that peak beta power was less than 10 times the mean power in the 0 to 100–Hz range (*20*, *62*). The tuning of synaptic weights was performed with synaptic and structural plasticity disabled; the firing rates thus represent initial values that can change once plasticity is enabled. The synaptic weights giving rise to this behavior are detailed in table S5.

#### Synaptic plasticity

The Graupner and Brunel (2012) plasticity model (*48*) has four free parameters per synapse $\gamma^{\mathrm{SYN},p}$ , $\gamma^{\mathrm{SYN},d}$ , $\theta^{\mathrm{SYN},p}$ , and $\theta^{\mathrm{SYN},d}$ , and our computational model contains four distinct types of plastic synapses SYN∈{CS,GS,SG,GG} . It has been observed experimentally that plasticity at CTX-to-STN synapses and GPe-to-STN synapses is heterosynaptic: Activity at CTX-to-STN synapses can influence the strength of GPe-to-STN synapses (*23*). For simplicity, we assumed that the same mechanism is present between STN-to-GPe synapses and GPe-to–GPe synapses. Although no experimental evidence is available to prove or disprove this, heterosynaptic plasticity is observed in many neuron types (*51*).

To model this heterosynaptic effect, the potentiation, $\theta^{\text{SYN},p}$ , and depression thresholds, $\theta^{\text{SYN},d}$ were set equal for excitatory and inhibitory synapses on each neuron. This results in a single potentiation threshold for the STN $\theta^{\text{STN},p}=\theta^{\text{CS},p}=\theta^{\text{GS},p}$ , and for the GPe $\theta^{\text{GPe},p}=\theta^{\text{SG},p}=\theta^{\text{GG},p}$ , and a single depression threshold for the STN $\theta^{\text{STN},d}=\theta^{\text{CS},d}=\theta^{\text{GS},d}$ and for the GPe $\theta^{\text{GPe},d}=\theta^{\text{SG},d}=\theta^{\text{GG},d}$ . An additional constraint necessary to reproduce plasticity behavior observed in the STN is that potentiation occurs at a higher calcium level compared to depression (*50*), so $\theta^{\text{STN},p}>\theta^{\text{STN},d}$ , and similarly it was assumed that $\theta^{\text{GPe},p}>\theta^{\text{GPe},d}$ . For simplicity, the same potentiation and depression rates were used for all synapses, $\gamma^{p}=\gamma^{\text{CS},p}=\gamma^{\text{GS},p}=\gamma^{\text{SG},p}=\gamma^{\text{GG},p}$ and $\gamma^{d}=\gamma^{\text{CS},d}=\gamma^{\text{GS},d}=\gamma^{\text{SG},d}=\gamma^{\text{GG},d}$ . Potentiation and depression thresholds were determined through an iterative trial-and-error process to balance long-term stability and responsiveness to excitatory inputs. Plasticity rules based on correlations between pre- and postsynaptic activity are inherently unstable as they induce a positive feedback loop whereby correlated activity at a synapse strengthens that synapse, increasing the likelihood that future activity will be correlated and leading to a state where all synaptic efficacies are at their maximum value (*79*). In the Graupner and Brunel model, the trade-off between sensitivity to correlations and stability of $\rho^{\mathrm{SYN}}$ can be controlled by adjusting $\theta^{\text{SYN},p},\theta^{\text{SYN},d},$ and $\gamma^{p}$ . The depression rate can be used with the plasticity time constant to control the rate at which plastic changes occur. If the plasticity is too sensitive, then all weights will move toward their attractor at $\rho^{\text{SYN}}=1$ . Conversely, if the plasticity is too stable, then it will not respond to any increases in activity or correlation among presynaptic neurons. As observed in Chu *et al.* (*23*), a bursting protocol consisting of 300 ms of 50-Hz firing repeated at 0.2 Hz, among all presynaptic cortical afferents, should potentiate CTX-to-STN synapses and GPe-to-STN synapses to double their initial conductance. No similar protocols were found in the literature to quantify the efficacy of STN-to-GPe synapses or GPe-to-GPe synapses, so it was assumed that this protocol would result in potentiation of GPe afferents. Future experiments applying such protocols to the GPe could yield information on whether this assumption is valid. It was desired that the mean value of $\rho^{\text{SYN}}$ for each synapse should be close to 0.33, to reproduce the effects observed in experiments for the STN where conductance of afferent cortical and pallidal synapses could be reduced by 50% , which can occur when $\rho^{\text{SYN}}=0$ , or increased by 100%, which can occur when $\rho^{\mathrm{SYN}}=1$ (*23*, *50*). Initial values for potentiation and depression thresholds were chosen based on the distribution of calcium concentrations within each segment; these values were then adjusted, along with the potentiation and depression rates, to achieve stable and responsive plasticity. The set of parameters defining the plasticity mechanism may be found in table S6.

#### Structural plasticity

The linear Butz and Van Ooyen model has two parameters governing the structural plasticity in each neuron, the target calcium concentrations ${\text{Ca}}^{\text{STN},L^{*}}$ and ${\text{Ca}}^{\text{GPe},L^{*}}$ , and the time constants for dendritic element growth $\tau^{\text{STN},z}$ and $\tau^{\text{GPe},z}$ . The time constants were set equal $\tau^{\text{STN},z}=\tau^{\text{GPe},z}=1800\;s$ , and the target calcium concentrations were set to the steady-state value determined in simulations with structural plasticity disabled. These values are reported in table S6.

### Simulation details

The model was implemented using C++ and the GPU parallelization library CUDA. A CUDA kernel was written to solve the system of equations governing the conductance-based neurons and the synapse dynamics. This allowed the system to be solved in parallel, thus substantially decreasing the computational time required for large, long-duration simulations. The conductance-based models are solved using the “Strang splitting” method, which has been demonstrated to preserve the correct limit cycle behavior in Hodgkin-Huxley type neurons while allowing the use of larger time step (*80*), and the synaptic equations were solved using the forward Euler method. To reduce the computational overhead associated with sending data to and from the GPU, a large number of iterations were solved continuously before control was returned to the CPU. Typically, 6400 iterations were used, corresponding to 200 ms of simulated time with the default time step of 0.03125 ms. CPU-bound code was responsible for initializing the simulation, generating stochastic spikes of cortical and striatal afferents, performing the updates to network connectivity that arise from the structural plasticity algorithm, and saving results. Custom scripts were written in Python to manage the model’s initialization and running, as well as perform data processing. Simulations typically required 3.5 s of real time for 1 s of simulated time using an Nvidia 3060ti GPU (Nvidia Corporation, Santa Clara, CA) and an Intel i7-9700k CPU (Intel Corporation, Santa Clara, CA). Simulations were run on the UCD Sonic high-performance computing cluster, and Stanford University’s Sherlock computing cluster. Further simulations were run on a local machine with an NVIDIA-sponsored Titan V GPU and Intel Core i9-10980XE CPU with 3.00 GHz.

Networks were initialized as follows: The average in-degree (number of incoming connections per postsynaptic neuron) of each synapse type was initialized to 10. Pre- and postsynaptic neurons were paired randomly according to a uniform distribution until this average in-degree was met. Membrane potentials were initialized between −80 and 50 mV, and gating variables for neurons and synapses were initialized between zero and one using a uniform random distribution. To generate stationary networks, we ran simulations for 13 hours of simulated physiological time, starting with the default cortical and iMSN activity described in Table 2 (Healthy). Corresponding data for the last hour of these simulations are shown as pre-input change conditions in Figs. 5 and 6. Then, step-like changes of cortical and iMSN inputs were performed, and simulations were continued for the simulated times shown in Figs. 5 and 6.

### Data evaluation

#### Beta power calculation

The instantaneous population firing rates were estimated by binning spike times into 1-ms intervals and averaging the number of spikes across the population at each interval. The power spectrum of the mean population spike train was then calculated using Welch’s method, with a window size of 1024 samples and 50% window overlap for spike train samples lasting 60 s. The integral beta power was estimated by integrating the power spectral density between 13 and 30 Hz.

#### Statistical analysis

We ran simulations for 10 random realizations of initial conditions for voltage, gating, and synaptic variables, as well as synaptic connectivity, noise, and cortical and iMSN (inhomogeneous) Poisson inputs, for each condition. Figures 5 and 6 show the averages of these 10 simulations (thick-colored curves) and the results of individual simulations (thin-colored curves).

#### Research standards

The parameters of our computational model were tuned to reflect the firing rates and neural activity observed in the dopamine-intact STN-GPe circuit of the rat (*52*, *53*, *77*, *78*). Synaptic and structural plasticity mechanisms were selected and parameterized to reproduce long term dynamics that have been observed in animal models of PD (*23*, *25*, *27*, *28*, *50*). The “Model fitting procedure” section of Materials and Methods describes the process in more detail.
