# Supplementary material for: Calcium-based synaptic and structural plasticity link pathological activity to synaptic reorganization in Parkinson’s disease
Source: Sci Adv. 2025 Nov 7;11(45):eadw7421. doi: 10.1126/sciadv.adw7421 (PMC12594202; doi:10.1126/sciadv.adw7421)
Supplement: Supplementary file 1 — Figs. S1 to S10 Tables S1 to S6 [file sciadv.adw7421_sm.pdf]

Science Advances
AAAS

Supplementary Materials for

Calcium-based synaptic and structural plasticity link pathological activity to synaptic reorganization in Parkinson's disease

Cathal McLoughlin *et al.*

Corresponding author: Peter A. Tass, ptass@stanford.edu

Sci. Adv. **11**, eadw7421 (2025)
DOI: 10.1126/sciadv.adw7421

This PDF file includes:

Figs. S1 to S10
Tables S1 to S6

Supplementary Figures

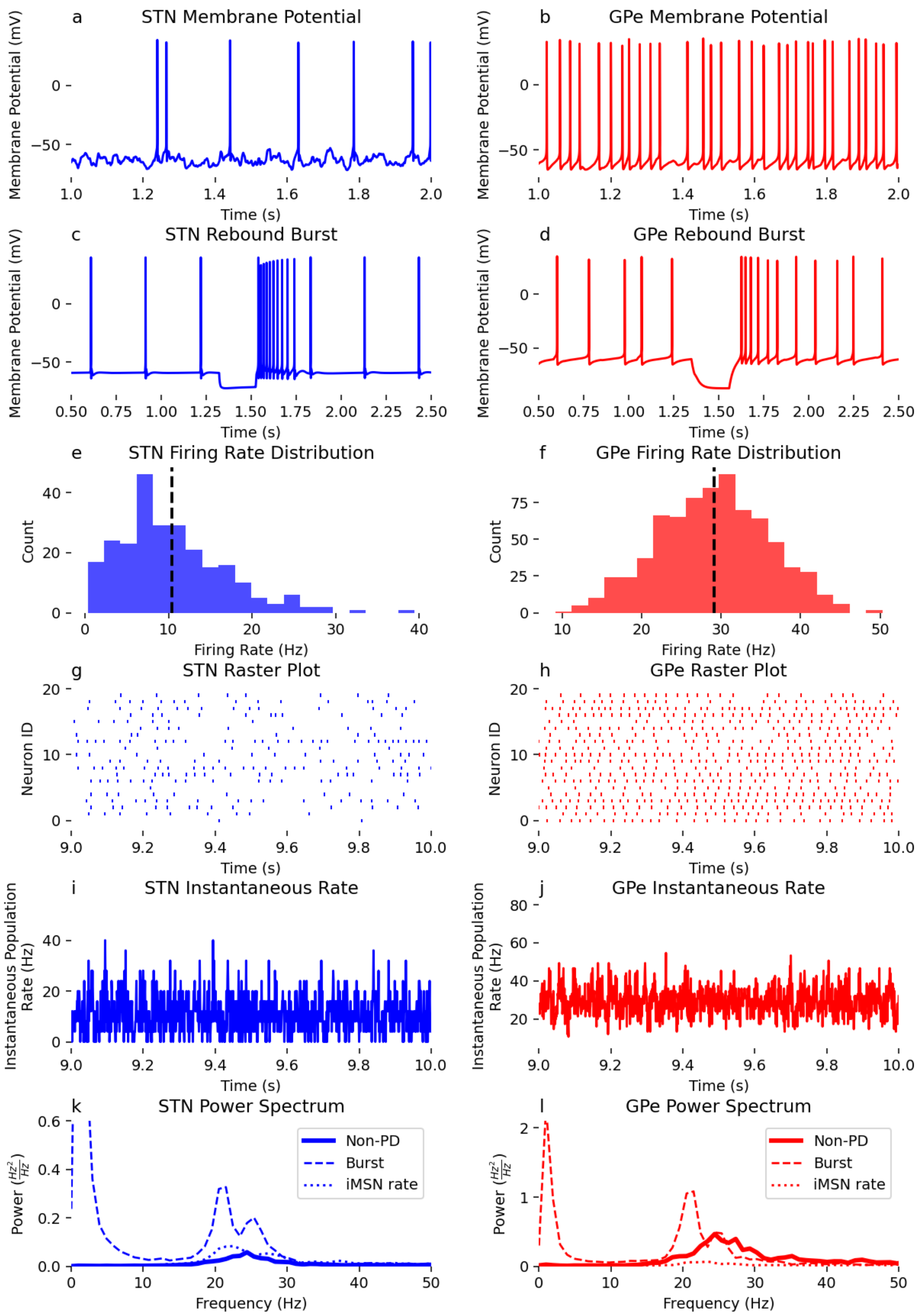

Figure S1: Baseline activity in the healthy state for STN and GPe populations. a,b: example membrane potentials for STN (a) and GPe (b) neurons. c,d: demonstration of rebound bursting in STN (c) and GPe (d) neurons. e,f histograms showing the distribution of firing rates in the STN (e) and GPe (f). g,h raster plots showing spiking activity in the STN (g) and GPe (h). i,j plots of population average instantaneous firing rates for the STN (i) and GPe (g) with 1 ms bin widths. k,l power spectra of the population average instantaneous firing rates in the healthy (Non-PD), PD burst (Burst), and hyperactive iMSNs (iMSN rate) states in the STN (k) and GPe (l).

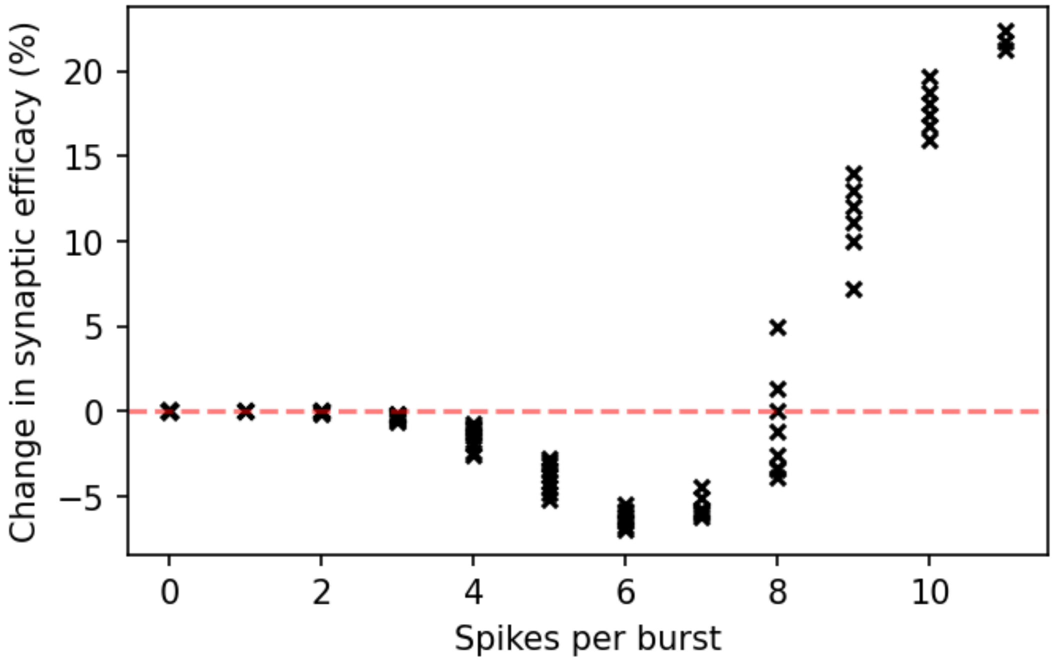

Figure S2: Acute change in synaptic efficacy versus spikes per rebound burst following a single STN hyperpolarization. The relationship between spikes per burst and change in synaptic efficacy for STN neurons, demonstrating that STN synapses are capable of being depressed, potentiated, or unchanged depending on the intensity of rebound bursts. The simulation used to produce this figure consisted of a single STN neuron which received inhibitory current injections of different strengths. Stronger inhibition produced rebound bursts with more spikes. Simulation conditions were as in Figure 4 in the main text, with all incoming and outgoing synaptic activity blocked. GPe to STN synaptic efficacies were initialized with a mean value of 0.33 and a standard deviation of 0.03. Simulations were run for 5 seconds. Changes in synaptic efficacy were calculated by comparing the initial and final values of synaptic efficacy following current injection. Twenty evenly spaced values of current strengths were used ranging from -1.0 to -4.2 $\frac{\mu A}{cm^2}$.

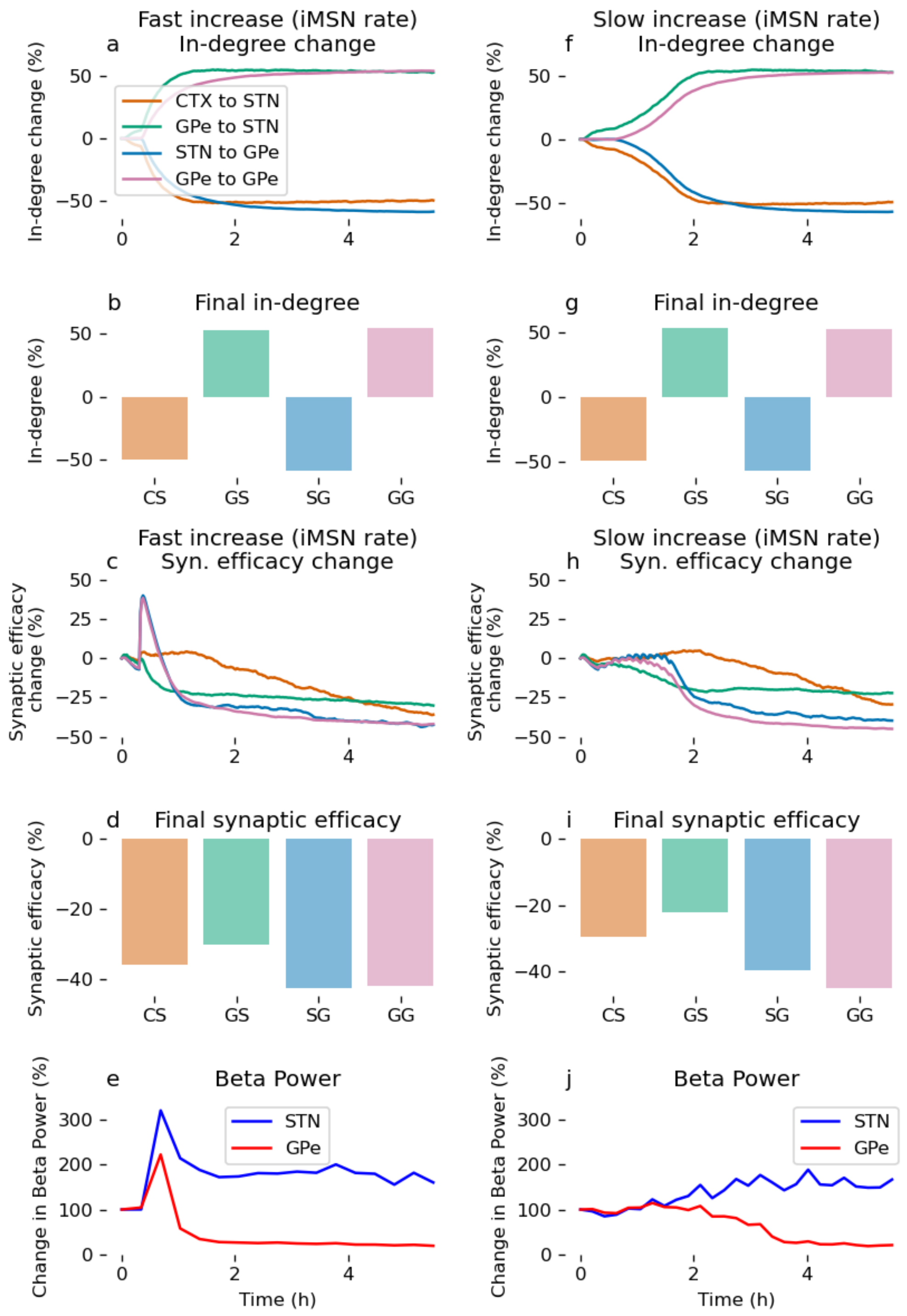

Figure S3: After transient differences subside, both fast and slow transitions to hyperactive iMSNs result in similar synaptic reorganization and beta power. Comparison of fast (a,b,c,d,e) and slow (f,g,h,I,j) changes in cortical and striatal activity due to a transition to hyperactive iMSNs (iMSN firing rate of 10Hz). In the fast condition, the transition was instantaneously. In the slow condition, the iMSN firing rate was linearly increased over 2 hours. The plasticity time constant was increased to 5 times its default value to allow faster convergence. a,f the change in average in-degree over time for each synapse type. b,g final distribution of in-degrees for each synapse type. c,h change in synaptic efficacy over time for each synapse type. d,i final distribution of synaptic efficacies for each synapse type. e,j change in beta power over time for the STN (blue) and GPe (red).

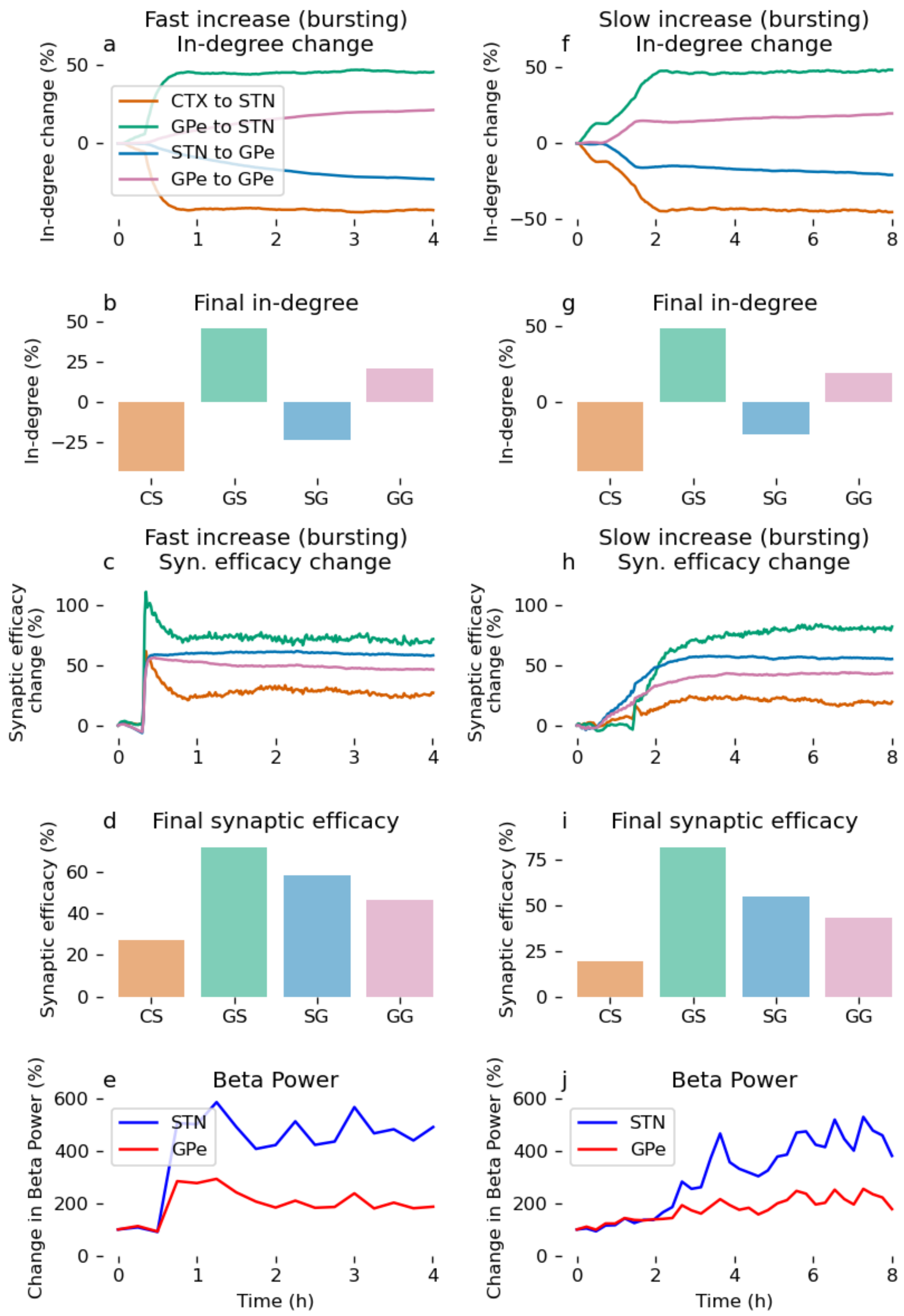

Figure S4: After transient differences subside, both fast and slow transitions to low-frequency bursting in cortical and iMSN inputs result in similar synaptic reorganization and beta power. Comparison of fast (a,b,c,d,e) and slow (f,g,h,I,j) changes in cortical and striatal activity under bursting conditions. In the fast condition, bursting activity was switched on instantaneously. The plasticity time constant was increased to 5 times its default value to allow faster convergence. In the slow condition, bursting activity was linearly increased over the course of 2 hours. a,f the change in average in-degree over time for each synapse type. b,g final distribution of in-degrees for each synapse type. c,h change in synaptic efficacy over time for each synapse type. d,i final distribution of synaptic efficacies for each synapse type. e,j change in beta power over time for the STN (blue) and GPe (red).

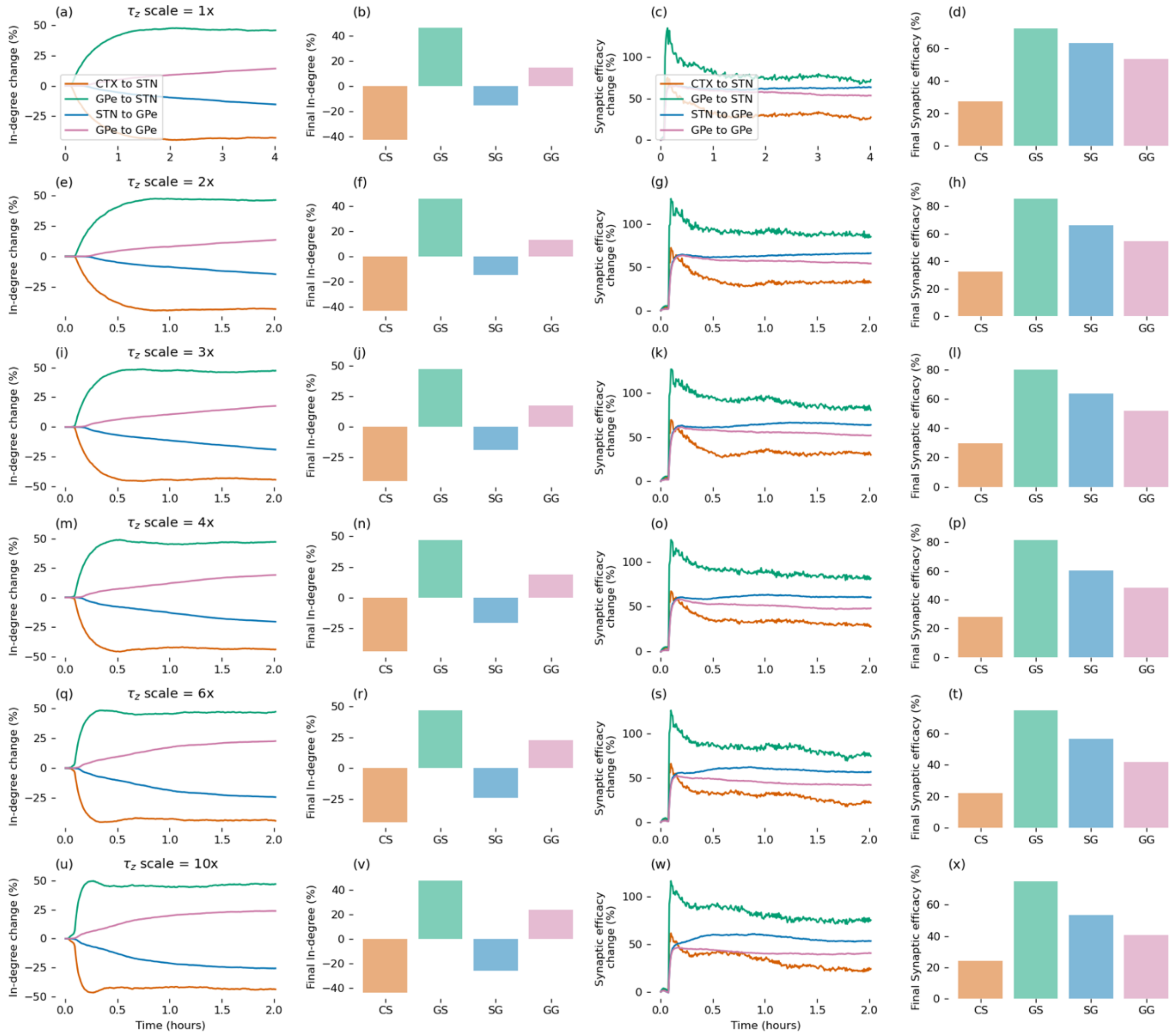

Figure S5: Comparison of cortical and iMSN bursting simulations with different values for element growth rate ($\boldsymbol{\tau_z}$). Simulations were performed using a network which had settled to a steady state structure under healthy activity. Subfigures a-d show the changes in degree over time (a), the final mean in degree changes (b), the changes in synaptic efficacy over time (c) and the final synaptic efficacy changes (d) for the default scaling of $\boldsymbol{\tau_z}$ = 1800s. Subsequent subfigures show these data for 2x, 3x, 4x, 6x, 10x faster time scales.

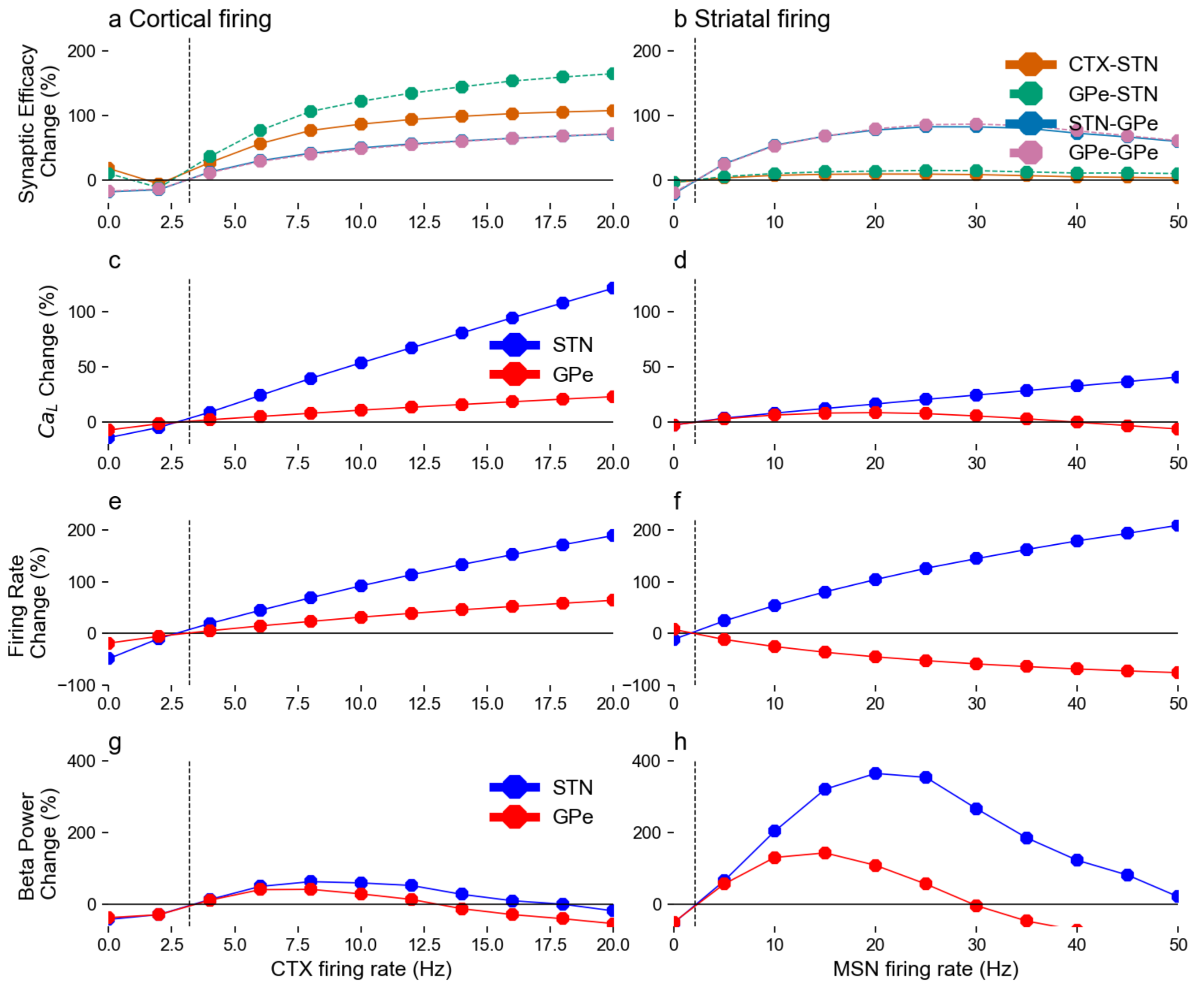

Figure S6: Examining the effects of cortical and striatal firing rates on STN-GPe circuit properties, with synaptic and structural plasticity disabled. A simulation was run for 12 hours to reach steady state with synaptic and structural plasticity enabled. The resulting network was then used as the initial state for different scenarios consisting of various cortical and iMSN firing rate changes. Cortical and striatal firing rates were varied independently. (a,b) Relationship between cortical and striatal firing rates and the change in efficacy at each synapse. (c,d) Relationship between cortical and striatal firing rates and population averaged long-term calcium concentrations Ca_L. (e,f) Relationship between cortical and striatal firing rates and STN and GPe mean firing rates. Results are presented as the percentage change relative to their values in the steady state that was used as initial state for the scenarios. Black dashed vertical lines denote the steady state firing rates in the cortical (3.2 Hz) and striatal (2.1 Hz) populations.

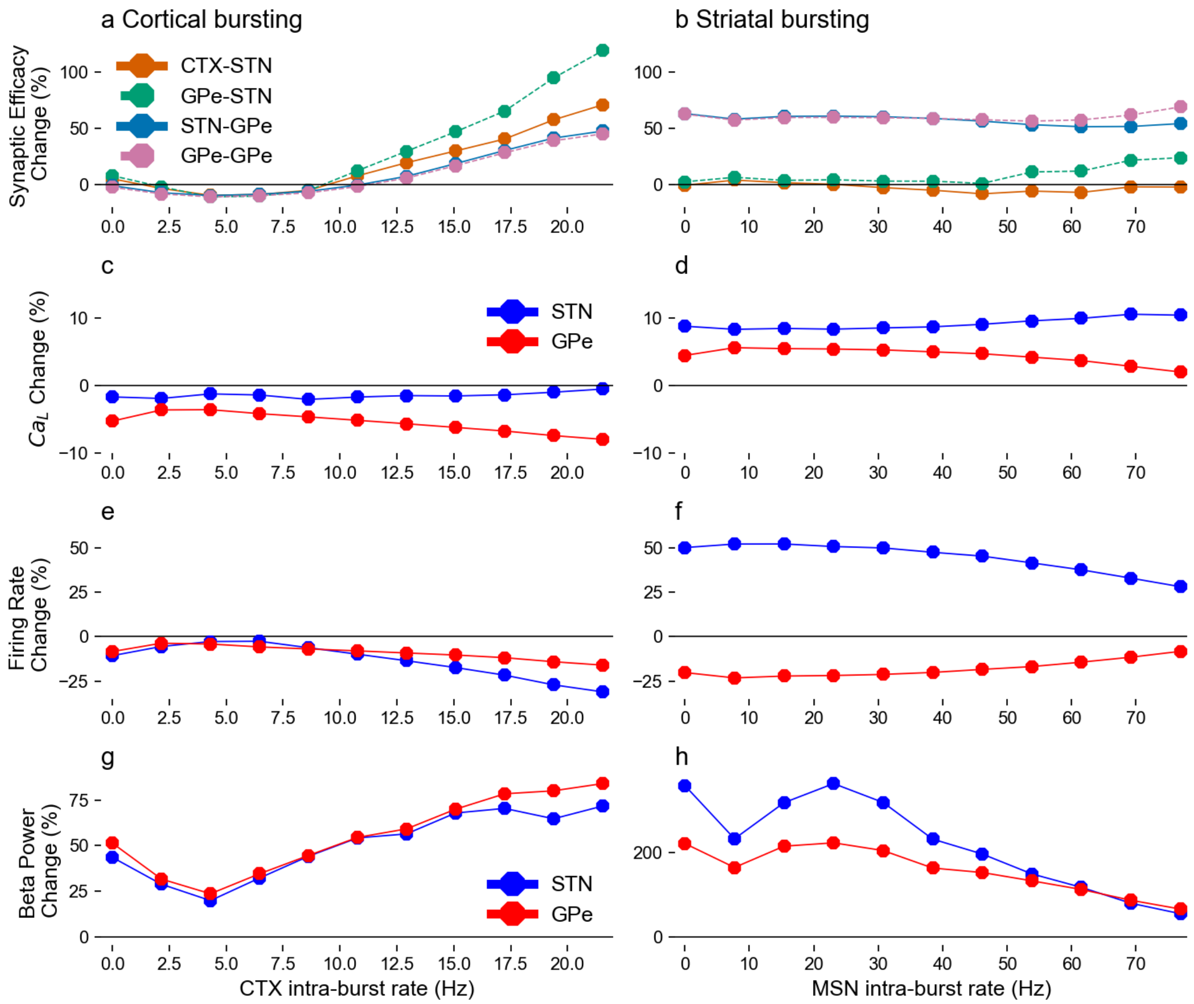

Figure S7: Examining the effects of cortical and iMSN intra-burst firing rates on STN-GPe circuit properties, in the absence of structural plasticity. The purpose of this experiment was to determine the effect of burst intensity, defined here as the density of spikes within a burst, on synaptic properties, beta power and firing rate. The mean firing rates and burst properties are the same as in the bursting state, with the values given in Table 2 in the main text. As the intra-burst firing rate was increased, correspondingly the firing rate during inter-burst intervals was decreased in order to maintain the mean firing rate of the population. Mean cortical and striatal firing rates were maintained at 2.8 Hz and 10 Hz respectively. Results are presented as the percentage change relative to that observed in the healthy state. Because the mean firing rates change in addition to the intra-burst firing rates, values of relative change are not guaranteed to cross zero on y axis. (a,b) Relationship between cortical and striatal intra-burst rate and the change in efficacy at each synapse. (c,d) Relationship between cortical and striatal intra-burst rate and population-averaged long-term calcium concentrations Ca_L. (e,f) Relationship between cortical and striatal intra-burst rate and the firing rate change within the STN and GPe.

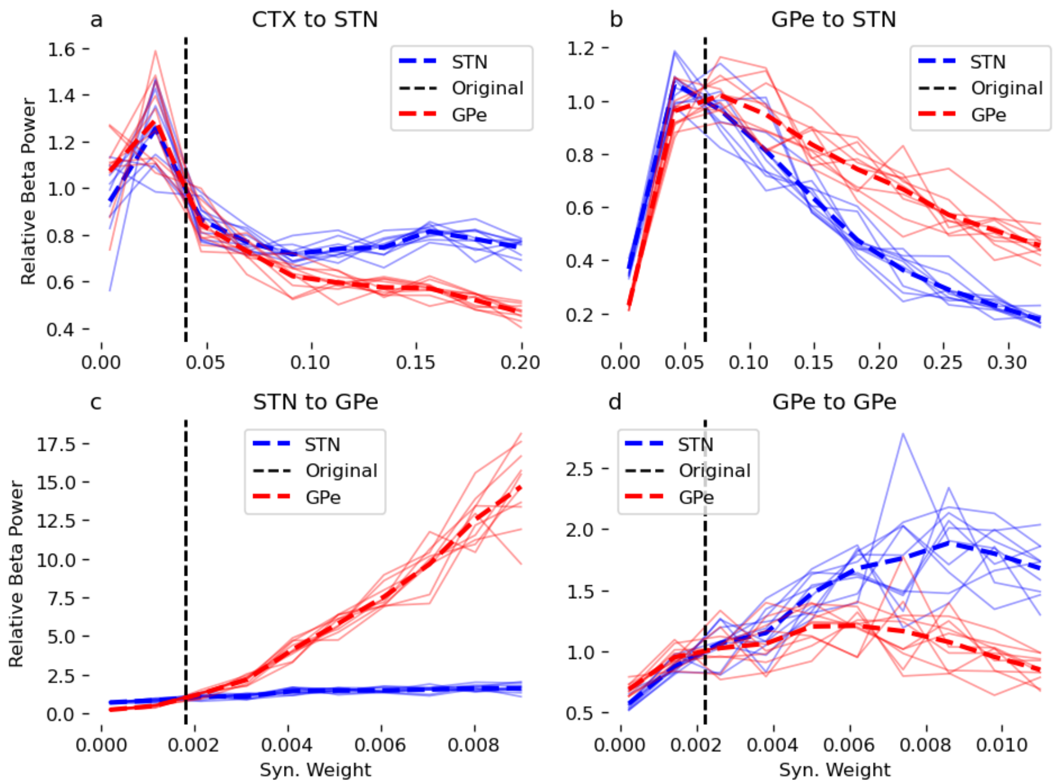

Figure S8: Effect of synaptic strengths on beta power within the STN and GPe healthy state with default activity. Synaptic and structural plasticity were disabled, and mean synaptic strengths were varied over a range of values. Beta power of the instantaneous average firing rate is reported on the y-axis. 10 simulations were performed for each of the 10 values within the range of the synaptic weights. Beta power in both the STN and GPe appears to generally decrease as the number of CTX to STN (a) and GPe to STN (b) connections are increased. Beta power in the STN and GPe appears to generally increase as the number of STN to GPe (c) and GPe to GPe (d) connections are increased.

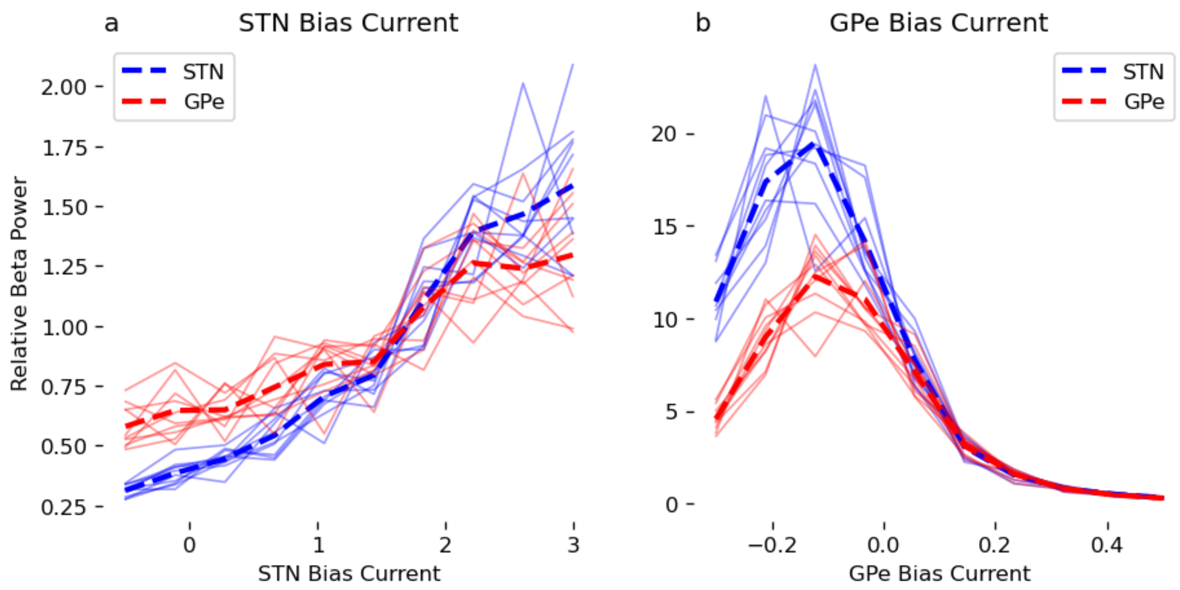

Figure S9: Effect of bias currents on beta power within the STN and GPe healthy state with default activity. Synaptic and structural plasticity were disabled, and bias currents to either the STN or GPe were varied. All neurons have the same bias current value. Beta power of the instantaneous average firing rate is reported on the y-axis. 10 simulations were performed for each of the 10 values within the range of bias currents. Increasing the STN bias current (a) or decreasing the GPe bias (b) leads to elevated beta power.

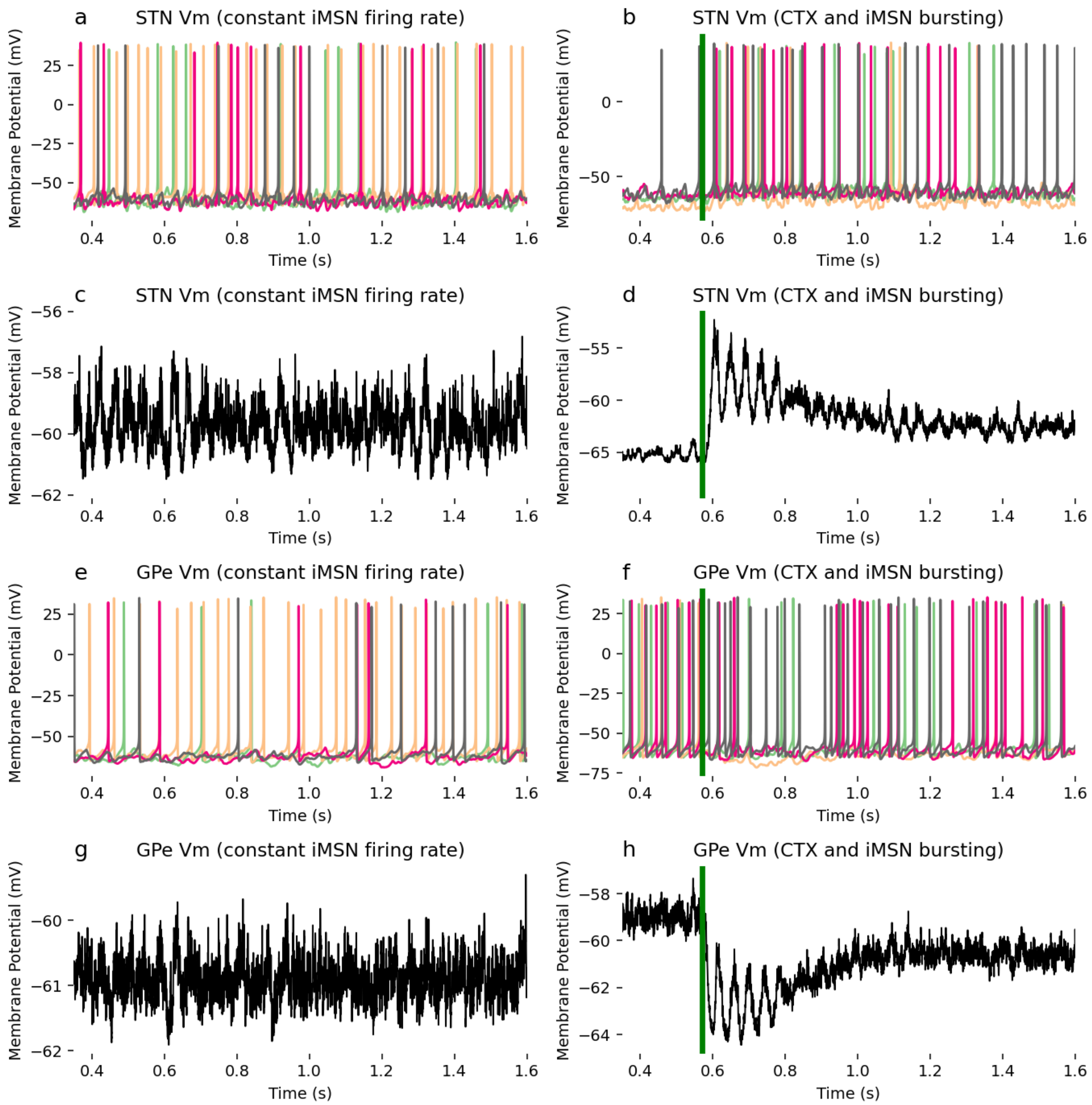

Figure S10: Sample membrane potential traces of STN and GPe neurons (colored traces) under hyperactive iMSNs condition (constant iMSN firing rate, a,e) and PD burst condition (CTX and iMSN bursting, b,f). Average membrane potentials across the entire STN (c,d) and GPe (g,h) are shown. The green vertical bar in panels b,d,f, and h marks the onset of a collective burst in iMSN and CTX inputs. Recordings were taken from 5 seconds of simulation following 60 hours of activity from simulations in Figure 5 of the main text.

Supplementary Tables

Table S1: STN gating parameters from Hahn and McIntyre (2010)

Parameter	Values	Parameter	Values	Parameter	Values
$\boldsymbol{C_m}$	1 µF/cm^2	θ_r	0.17e-3 mM	τ_r	2 ms
$\boldsymbol{L}$	60 µm	k_a	-14.7 mV	θ_a^τ	-40 mV
$\boldsymbol{Diam}$	60 µm	k_b	7.5 mV	$\theta_b^{\tau1}, \theta_b^{\tau2}$	-60, -40 mV
$\boldsymbol{g_l}$	0.35 mS/cm^2	k_c	-5 mV	$\theta_c^{\tau1}, \theta_c^{\tau2}$	-27, 50 mV
$\boldsymbol{g_{Na}}$	49 mS/cm^2	k_{d_1}	7.5 mV	$\theta_{d_1}^{\tau1}, \theta_{d_1}^{\tau2}$	-40, -20 mV
$\boldsymbol{g_K}$	57 mS/cm^2	k_{d_2}	0.02 µM	$\theta_h^{\tau1}, \theta_h^{\tau2}$	-50, -50 mV
$\boldsymbol{g_A}$	5 mS/cm^2	k_h	6.4 mV	θ_m^τ	-53 mV
$\boldsymbol{g_L}$	15 mS/cm^2	k_m	-8 mV	$\theta_n^{\tau1}, \theta_n^{\tau2}$	-40, -40 mV
$\boldsymbol{g_T}$	5 mS/cm^2	k_n	-14 mV	$\theta_p^{\tau1}, \theta_p^{\tau2}$	-27, -102 mV
$\boldsymbol{g_{Ca-K}}$	1 mS/cm^2	k_p	-6.7 mV	$\theta_q^{\tau1}, \theta_q^{\tau2}$	-50, -50 mV
$\boldsymbol{E_l}$	-60 mV	k_q	5.8 mV	σ_a	-0.5 mV
$\boldsymbol{\theta_a}$	-45 mV	k_r	-0.08 µM	σ_b^1, σ_b^2	-30, 10 mV
$\boldsymbol{\theta_b}$	-90 mV	τ_a^0, τ_a^1	1, 1 ms	σ_c^1, σ_c^2	-20, 15 mV
$\boldsymbol{\theta_c}$	-30.6 mV	τ_b^0, τ_b^1	0, 200 ms	$\sigma_{d_1}^1, \sigma_{d_1}^2$	-15, 20 mV
$\boldsymbol{\theta_{d_1}}$	-60 mV	τ_c^0, τ_c^1	45, 10 ms	σ_h^1, σ_h^2	-15, 16 mV
$\boldsymbol{\theta_{d_2}}$	0.1 µM	$\tau_{d_1}^0, \tau_{d_1}^1$	400, 500 ms	σ_m	-0.7 mV
$\boldsymbol{\theta_h}$	-45.5 mV	τ_h^0, τ_h^1	0, 24.5 ms	σ_n^1, σ_n^2	-40, 50 mV
$\boldsymbol{\theta_m}$	-40 mV	τ_m^0, τ_m^1	0.2, 3 ms	σ_p^1, σ_p^2	-10, 15 mV
$\boldsymbol{\theta_n}$	-41 mV	τ_n^0, τ_n^1	0, 11 ms	σ_q^1, σ_q^2	-15, 16 mV
$\boldsymbol{\theta_p}$	-56 mV	τ_p^0, τ_p^1	5, 0.33 ms	I_{bias}	-1.0 nA
$\boldsymbol{\theta_q}$	-85 mV	τ_q^0, τ_q^1	0, 400 ms		

Table S2: GPe gating parameters from Hahn and McIntyre (2010)

Parameter	Values	Parameter	Values	Parameter	Values
C_m	1 µF/cm^2	θ_r	0.17e-3 mM	τ_r	2 ms
L	60 µm	k_a	-14.7 mV	θ_a^τ	-40 mV
$Diam$	60 µm	k_b	7.5 mV	$\theta_b^{\tau 1}, \theta_b^{\tau 2}$	-60, -40 mV
g_l	0.35 mS/cm^2	k_c	-5 mV	$\theta_c^{\tau 1}, \theta_c^{\tau 2}$	-27, 50 mV
g_{Na}	49 mS/cm^2	k_{d_1}	7.5 mV	$\theta_{d_1}^{\tau 1}, \theta_{d_1}^{\tau 2}$	-40, -20 mV
g_K	57 mS/cm^2	k_{d_2}	0.02 µM	$\theta_h^{\tau 1}, \theta_h^{\tau 2}$	-50, -50 mV
g_A	5 mS/cm^2	k_h	6.4 mV	θ_m^τ	-53 mV
g_L	15 mS/cm^2	k_m	-8 mV	$\theta_n^{\tau 1}, \theta_n^{\tau 2}$	-40, -40 mV
g_T	5 mS/cm^2	k_n	-14 mV	$\theta_p^{\tau 1}, \theta_p^{\tau 2}$	-27, -102 mV
g_{Ca-K}	1 mS/cm^2	k_p	-6.7 mV	$\theta_q^{\tau 1}, \theta_q^{\tau 2}$	-50, -50 mV
E_l	-60 mV	k_q	5.8 mV	σ_a	-0.5 mV
θ_a	-45 mV	k_r	-0.08 µM	σ_b^1, σ_b^2	-30, 10 mV
θ_b	-90 mV	τ_a^0, τ_a^1	1, 1 ms	σ_c^1, σ_c^2	-20, 15 mV
θ_c	-30.6 mV	τ_b^0, τ_b^1	0, 200 ms	$\sigma_{d_1}^1, \sigma_{d_1}^2$	-15, 20 mV
θ_{d_1}	-60 mV	τ_c^0, τ_c^1	45, 10 ms	σ_h^1, σ_h^2	-15, 16 mV
θ_{d_2}	0.1 µM	$\tau_{d_1}^0, \tau_{d_1}^1$	400, 500 ms	σ_m	-0.7 mV
θ_h	-45.5 mV	τ_h^0, τ_h^1	0, 24.5 ms	σ_n^1, σ_n^2	-40, 50 mV
θ_m	-40 mV	τ_m^0, τ_m^1	0.2, 3 ms	σ_p^1, σ_p^2	-10, 15 mV
θ_n	-41 mV	τ_n^0, τ_n^1	0, 11 ms	σ_q^1, σ_q^2	-15, 16 mV
θ_p	-56 mV	τ_p^0, τ_p^1	5, 0.33 ms	I_{bias}	-1.0 nA
θ_q	-85 mV	τ_q^0, τ_q^1	0, 400 ms		

Table S3: STN and GPe conductances

Conductance	STN (mS/cm^2)	GPe (mS/cm^2)
g^{Na}	49	40
g^{K}	57	4.2
g^{T}	4.0	0.134
$g^{\mathrm{Ca\text{-}K}}$	1.0	0.1
g^{leak}	0.35	0.04
g^{A}	5.0	
g^{L}	0.35	

Table S4: Parameters for AMPA, GABA, and NMDA synapses

Symbol	AMPA and GABA	NMDA
Φ^{y}	20.0	20.0
$\upsilon^{y,\mathrm{fast}}$	0.903	0.527
$\upsilon^{y,\mathrm{slow}}$	0.097	0.473
$\tau^{y,\mathrm{rise}}$	0.58	2.0
$\tau^{y,\mathrm{fast}}$	7.6	10.0
$\tau^{y,\mathrm{slow}}$	25.69	45.0

Table S5: Synaptic conductances and their minimal values. Corresponding maximal values are four times the minimal values.

Synapse	Value (mS cm^{-2})
$w^{(\mathrm{CTX\text{-}STN,min})}$	0.04
$w^{(\mathrm{GPe\text{-}STN,min})}$	0.065
$w^{(\mathrm{STN\text{-}GPe,min})}$	0.0018
$w^{(\mathrm{GPe\text{-}GPe,min})}$	0.0022
$w^{(\mathrm{MSN\text{-}GPe,min})}$	0.03

Table S6: Synaptic and structural plasticity parameters

Symbol	Value
$\theta^{(\mathrm{STN},p)}$	17.8 nM
$\theta^{(\mathrm{STN},d)}$	12.0 nM
$\theta^{(\mathrm{GPe},p)}$	0.29 nM
$\theta^{(\mathrm{GPe},d)}$	0.2 nM
γ^{p}	200 s^{-1}
γ^{d}	20 s^{-1}
$\mathrm{Ca}^{(\mathrm{STN},L*)}$	3.86 nM
$\mathrm{Ca}^{(\mathrm{GPe},L*)}$	0.058 nM
$\tau^{(\mathrm{STN},z)}$	300 s
$\tau^{(\mathrm{GPe},z)}$	300 s
